# Accurate and Flexible Single Cell to Spatial Transcriptome Mapping with Celloc

**DOI:** 10.1002/smsc.202400139

**Published:** 2024-06-26

**Authors:** Wang Yin, Xiaobin Wu, Linxi Chen, You Wan, Yuan Zhou

**Affiliations:** ^1^ Department of Biomedical Informatics School of Basic Medical Sciences Peking University 38 Xueyuan Road Beijing 100191 China; ^2^ State Key Laboratory of Vascular Homeostasis and Remodeling Peking University 38 Xueyuan Road Beijing 100191 China; ^3^ Department of Neurobiology School of Basic Medical Sciences Neuroscience Research Institute Peking University 38 Xueyuan Road Beijing 100191 China; ^4^ Provincial Key Laboratory of Research in Structure Birth Defect Disease Guangzhou Women and Children's Medical Center Guangzhou Medical University No. 9 Jinshui Road Guangzhou Guangdong 510623 China; ^5^ Key Laboratory of Carcinogenesis and Translational Research (Ministry of Education/Beijing) Peking University Cancer Hospital & Institute 52 Fucheng Road Beijing 100142 China

**Keywords:** graph attention autoencoders, single‐cell mapping, spatial transcriptomics

## Abstract

Accurate mapping between single‐cell RNA sequencing (scRNA‐seq) and low‐resolution spatial transcriptomics (ST) data compensates for both limited resolution of ST data and missing spatial information of scRNA‐seq. Celloc, a method developed for this purpose, incorporates a graph attention autoencoder and comprehensive loss functions to facilitate flexible single cell‐to‐spot mapping. This enables either the dissection of cell composition within each spot or the assignment of spatial locations for every cell in scRNA‐seq data. Celloc's performance is benchmarked on simulated ST data, demonstrating superior accuracy and robustness compared to state‐of‐the‐art methods. Evaluations on real datasets suggest that Celloc can reconstruct cellular spatial structures with various cell types across different tissues and histological regions.

## Graphical abstract

Celloc is a graph attention autoencoder and multiple loss‐based deep learning method that takes single‐cell RNA sequencing data and spatial transcriptome data as input, allowing to fill all spots with appropriate single cells (regular mapping) or allocate all single cells to their best‐matched spots (greedy mapping).

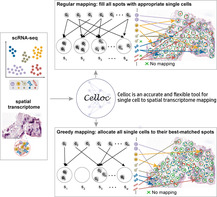

## 1 Introduction

A detailed depiction of the spatial expression patterns of heterogeneous cell populations in complex tissues is crucial, as single‐cell spatial organization is a key factor in determining cell status and function.^[^ ^1^, ^2^ ^]^ Spatial transcriptomics (ST) enables the measurement of gene expression across tissue sections, preserving the spatial context of cells and their interactions. However, many popular ST techniques have limitations in terms of resolution, sensitivity, or gene coverage.^[^ ^3^, ^4^, ^5^ ^]^ Single‐cell RNA sequencing (scRNA‐seq) can overcome these limitations by profiling the transcriptomes of individual cells, but it loses the spatial information during the tissue dissociation step.^[^ ^6^ ^]^ To address this issue, several computational methods, such as Cell2location,^[^ ^7^ ^]^ CARD,^[^ ^8^ ^]^ SpatialDWLS,^[^ ^9^ ^]^ RCTD,^[^ ^10^ ^]^ and our previous tool SpatialcoGCN,^[^ ^11^ ^]^ have been developed to deconvolute ST spots into proportions of different cell types. Nonetheless, these methods only estimate the proportions of cell types in each spatial spot but cannot achieve single‐cell resolution. Therefore, Redeconve^[^ ^12^ ^]^ treats every cell in scRNA‐seq as a single‐cell state and deconvolutes ST data into composition of single‐cell states. SpatialScope^[^ ^13^ ^]^ generates pseudosingle cells by leveraging deep generative models and matches pseudosingle cells to the estimated cell type composition per spot to reach the single‐cell resolution. Nonetheless, the objective of all of these spatial deconvolution methods is to infer the cell type composition for each spot, and it cannot achieve exact single cell‐to‐spot mapping, hindering the discovery of spatially defined cell states, their interaction patterns, and their surrounding communities.^[^ ^14^ ^]^ Therefore, there is an urgent need for computational methods that can achieve high‐resolution scRNA‐seq and ST data linking to reconstruct the spatial organization of each single cell.

To address the limitations of the spatial deconvolution approach, recent methods such as CytoSPACE,^[^ ^15^ ^]^ Tangram,^[^ ^16^ ^]^ and CellTrek^[^ ^17^ ^]^ have been developed to directly establish the single cell‐to‐spot mapping relationships from scRNA‐seq to ST data. Although all methods minimize the correlation‐based cost function inferred from gene expressions between scRNA‐seq and ST data, their approaches and outcomes are different. Tangram focused on the consistency in cell density and gene expression, generating mapping probabilities from cells to spots. CytoSPACE achieves single cell‐to‐spot mapping based on the inferred cell counts per ST spot, cell type fractions, and corresponding gene expression. CellTrek uses spatial Seurat^[^ ^18^ ^]^ to identify shared embedding between scRNA‐seq and ST data and then applies random forest modeling to predict spatial coordinates of each single cell. Given the importance of dissecting cell composition of each spot in ST data, a more accurate method for (regular) single cell‐to‐spot mapping is continuously required.

Besides, we also note that the previous methods are often conservative when performing the single cell‐to‐spot mapping, allowing only part of the cells in scRNA‐seq data mapped to the spots. As current scRNA‐seq techniques like the popular 10× Genomics Chromium platform can capture a big single‐cell atlas covering several thousands of cells, a greedy mapping of every single cell to the spatial coordinates is also deemed useful to comprehensively interpret the spatial pattern and interactions among single cells. Such greedy mapping can assign a spatial location to each single cell in the ST data. However, it is noteworthy the objective of greedy mapping is different from CeLEry^[^ ^19^ ^]^ that aims at *de novo* prediction of spatial location of single cells. In the training phase, CeLEry learns relationships between gene expression and (relative) cell location from ST data. But when exerting the prediction, ST data is no longer considered, and the predicted spatial coordinates of cells are no longer aligned to the ST data. In other words, there is no exact single cell‐to‐spot mapping between single cells and the reference ST data in CeLEry's results. In contrast, in greedy mapping, the spatial coordinates of cells totally depend on the spatial coordinates of their mapped spots in the ST data, enabling direct linking between single cells and the reference ST spots.

Here, we find that both regular and greedy single cell‐to‐spot mapping can be achieved under the same computational framework without significant modifications to the algorithm. Specifically, we developed Celloc, a graph attention autoencoder (GATE) and multiple loss‐based method that allows either dissecting cell composition of each spot (regular mapping) or predicting spatial location of every cell in scRNA‐seq data (greedy mapping), enabling a more flexible and straightforward investigation of single‐cell data with spatial topography. Celloc exploits graph autoencoder to properly encode single cells and spots into the embedding space. It also considers a comprehensive collection of loss terms including gene expression similarity, embedding correlation, and cell quantity per spot to generate high resolution, resulting in a quantitative mapping between scRNA‐seq and ST data. On both simulated and real ST datasets, we found that Celloc substantially outperformed the related methods for resolving single‐cell spatial distribution.

## 2 Results

### 2.1 Overview of Celloc

**Figure 1:**
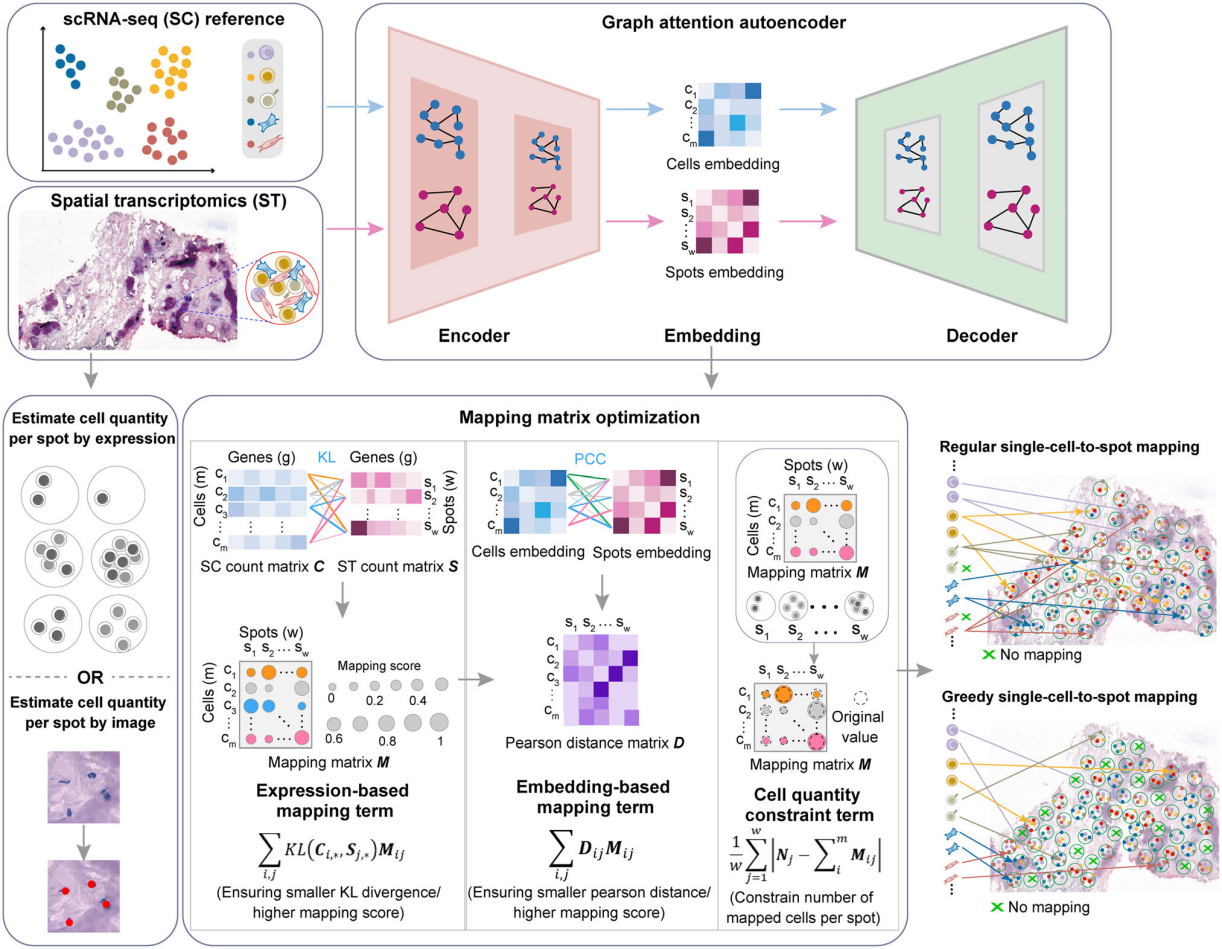
Schematic overview of Celloc. Celloc first respectively projected scRNA‐seq data and ST data into embedding space via the encoder architecture of GATE. Next, Celloc estimated the expected number of cells per ST spot using a method for estimating RNA abundance or high‐resolution H&E image nucleus segmentation. Then, Celloc optimized a comprehensive loss function that consists of three terms: the expression‐based mapping term; embedding‐based mapping term; and the cell quantity constraint term. The cell quantity constraint term is useful even in the greedy mapping task as it can prevent cells from aggregating into a very small fraction of spots. Finally, the mapping of single cells to spatial spots is completed by finding the best‐matched cell‐spot pairs from the solved mapping score matrix. Two mapping tasks are available in Celloc using nearly the same computational framework: 1) regular mapping, that is, filling ST spots with expected number of cells mapped from scRNA‐seq and 2) greedy mapping, that is., assigning every cell in scRNA‐seq data to its best‐matched spatial location.

Celloc is a method utilizing deep learning to map individual cells from a reference scRNA‐seq atlas to spatial locations in a ST dataset (**Figure** **1**). This is achieved by correlating expression profiles between cells and mapped spots, preserving spatial coherence of gene expression. Celloc employs a GATE to encode gene expressions and optimizes a multiple‐loss objective function to determine the mapping matrix. This allows Celloc to 1) enhance ST data resolution and gene expression quantity by filling spots with appropriate cells from scRNA‐seq (regular mapping) and 2) investigate spatial patterns across the entire scRNA‐seq dataset by assigning spatial locations to each cell (greedy mapping).

In Celloc, both regular and greedy single cell‐to‐spot mappings are accomplished within a unified computational framework (Figure 1). It involves four main steps. First, scRNA‐seq and ST data are projected into respective embedding spaces using the encoder architecture of GATE,^[^ ^20^ ^]^ retaining their structural and content information. GATE is a deep learning method that can learn low‐dimensional latent representations from high‐dimensional data. Second, Celloc estimates the expected number of cells per ST spot, utilizing accurate nucleus segmentation from high‐resolution hematoxylin and eosin staining (H&E) images or by estimating cell numbers through RNA abundance estimation methods. We employ StarDist,^[^ ^21^ ^]^ which has also been employed by SpatialScope,^[^ ^13^ ^]^ as the default tool for nucleus segmentation on H&E histological images. Third, a loss function incorporating multiple terms is optimized using the Adam optimizer, which contains three terms: the expression‐based mapping term ensuring that the cells and spots with smaller kullback‐leibler (KL) divergence have higher mapping scores; the embedding‐based mapping term ensuring that mapped cells and spots have high Pearson correlation coefficients (PCC) in the embedding space; and the cell quantity constraint term ensuring that the number of cells mapping to each spot is equal to estimated cells number per spot and increases the variance of mapping scores. The cell quantity constraint term is useful even in the greedy mapping task as it can prevent cells from aggregating into a very small fraction of spots. Finally, single cell‐to‐spot mapping is achieved by identifying the best‐matched cell spot pairs from the solved mapping score matrix (Figure S1, Supporting Information).

### 2.2 Benchmark Evaluation on Simulated ST Data

To test the performance of Celloc, we leveraged simulated Slide‐seq datasets of mouse cerebellum (11 cell types) and hippocampus (17 cell types) sections from CytoSPACE.^[^ ^15^ ^]^ Because the spatial resolution of ST data varies depending on the technology used, simulated ST data with an average of 5, 15, and 30 cells per spot can be obtained, respectively, by adjusting the bin size. For scRNA‐seq data, each Slide‐seq bead was replaced by the most correlated single‐cell expression profile of the same cell type derived from a scRNA‐seq atlas of the same brain region.^[^ ^22^ ^]^ To emulate technical variation between platforms, noise was added to the scRNA‐seq data in defined amounts. We perturbed 5%, 10%, and 25% of genes for this purpose (Experimental Section). The cerebellum and hippocampus scRNA‐seq datasets both have 23 372 cells.

**Figure 2:**
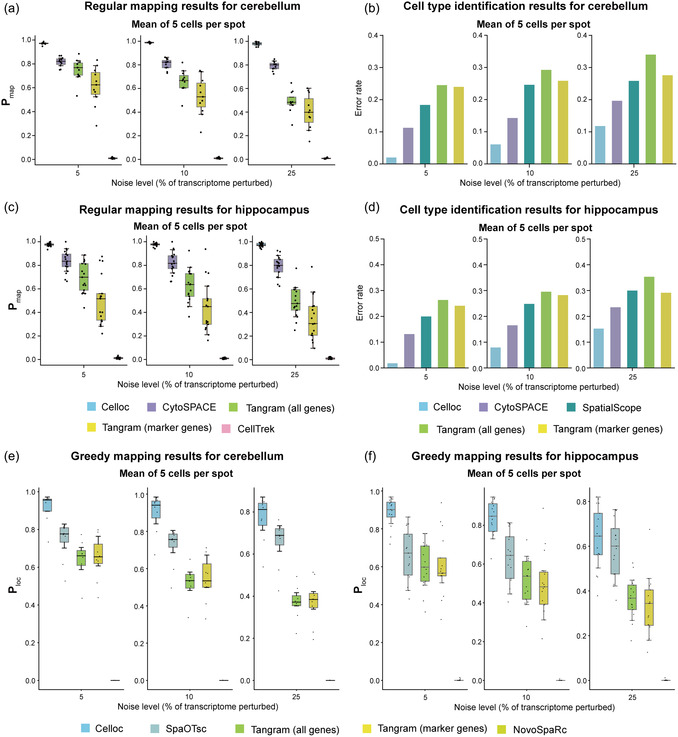
Evaluation of Celloc's performance on simulated cerebellum and hippocampus datasets. a) On the simulated cerebellum datasets, the performances of Celloc and the previous methods for filling ST spots with expected number of cells mapped from scRNA‐seq (regular mapping) across different noise levels were compared. Each point represents a single‐cell type (mouse cerebellum, *n* = 11). Boxplots: center line, median; box limits, upper and lower quartiles. The boxplots comparing the mapping precisions (Pmap). b) The barplots comparing cell type identification accuracy by calculating the cell type misclassification error rate on the simulated cerebellum datasets. c) The performances of Celloc and the previous methods for fregular mapping on the simulated hippocampus datasets. d) The barplots comparing cell type identification accuracy on the simulated hippocampus datasets. e,f) Evaluation of greedy mapping on simulated cerebellum and hippocampus datasets.

The performance for the regular single cell‐to‐spot mapping task was first benchmarked. We applied Celloc to the simulated ST datasets to solve the single cell composition of each spot in the simulated ST datasets. We compared Celloc with three recently described algorithms for regular single‐cell‐to‐spot mapping: CytoSPACE,^[^ ^15^ ^]^ Tangram,^[^ ^16^ ^]^ and CellTrek.^[^ ^17^ ^]^ We measured the accuracy of scRNA‐seq and ST mapping using single‐cell mapping precision ($P_{\text{map}}$), where a high $P_{\text{map}}$ indicates more accurate mapping. In addition, we compared the performances of Celloc, CytoSPACE, SpatialScope,^[^ ^13^ ^]^ Tangram, which are capable of inferring cell type labels at the single‐cell level. We assessed their cell type identification accuracy at the single‐cell resolution by calculating the misclassification error rate, which represents the proportion of cells with misclassified cell type labels. On both simulated and real ST datasets, we found that Celloc substantially outperformed the related methods (**Figure** **2**, and S2, Supporting Information).

For the simulated cerebellum dataset, across multiple evaluated noise levels and spot resolution, Celloc achieved substantially higher mapping accuracy than other methods. Across different simulation conditions, Celloc showed higher $P_{\text{map}}$ (Figure 2a and S2b, Supporting Information) and lower error rate (Figure 2b and S2b, Supporting Information) compared with other methods. As the noise level increased or the spot resolution decreased, the performance of other methods declined significantly, while Celloc's performance was relatively stable and kept higher than 60% $P_{\text{map}}$ in median. The visualization of the mapping result demonstrated that Celloc reconstructed the original spatial pattern of the simulated cerebellum data and showed the best performance compared with other methods (Figure S3a,b, Supporting Information). Celloc reconstructed accurate spatial patterns of granule cells, oligodendrocytes, Purkinje neurons, Bergmann glia, and choroid cells. SpatialScope, CytoSPACE, and Tangram could also reconstruct the spatial structure, but the smaller number of accurately mapped cells makes the spatial structure relatively ambiguous. CellTrek results show that some spots have no mapping results and other spots have too many mapped cells, which led to erroneous spatial structure predictions (Figure S3b, Supporting Information).

We observed similar results on the simulated hippocampus dataset. Celloc achieved substantially higher precision than other methods for mapping single cells to their known locations. Across different simulation conditions, Celloc showed higher $P_{\text{map}}$ (Figure 2c, and S2c, Supporting Information) and lower error rate (Figure 2d, and S2d, Supporting Information) compared with other methods. Celloc's performance was relatively stable as the noise level increased or the spot resolution decreased. The visualization of the mapping result demonstrated that Celloc reconstructed the original spatial pattern of the simulated hippocampus data and showed the best performance compared with other methods (Figure S4a,b, Supporting Information). Celloc reconstructed the accurate spatial structure of astrocyte, CA1, choroid cell, dentate gyrus, and polydendrocyte.

By summing up the number of mapped cell per cell type of each spot, Celloc could also estimate the cell type proportion of each spot like traditional deconvolution methods (Cell2location,^[^ ^7^ ^]^ CARD,^[^ ^8^ ^]^ SpatialDWLS,^[^ ^9^ ^]^ RCTD,^[^ ^10^ ^]^ and SpatialcoGCN^[^ ^11^ ^]^). We compare the performance of Celloc and traditional deconvolution methods on ST data deconvolution by calculating PCC, where a higher PCC value indicates better prediction accuracy. The results showed that Celloc performs significantly better than traditional deconvolution methods both on simulated cerebellum and on hippocampus datasets (Figure S5, Supporting Information).

We further compared the performance of Celloc, Tangram, SpaOTsc,^[^ ^23^ ^]^ and NovoSpaRc^[^ ^24^ ^]^ on the greedy single cell‐to‐spot mapping. Among the above regular mapping methods, only Celloc and Tangram can implement greedy mapping. But few other methods’ intermediate results could also contain information for the greedy mapping. For example, SpaOTsc aims at inferring cell–cell communications from scRNA‐seq data, employing an optimal transport algorithm to construct the spatial mapping of cells as an intermediate step. NovoSpaRc is another method that utilizes the optimal transform algorithm to implicitly map cells to tissue locations. By assuming cells in physical proximity also share similar gene expression profiles, NovoSpaRc can recover the spatial locations of scRNA‐seq cells without the reliance on a spatial reference. To ensure comparability, we used the spatial location information from the ST spots as the spatial reference for NovoSpaRc's spatial mapping. In order to evaluate the methods on greedy single cell‐to‐spot mapping task, we defined location prediction precision ($P_{\text{loc}}$), which represents the percentage of cells per cell type whose spatial locations were correctly assigned (Experimental Section). We only assessed greedy mapping under simulation condition of the mean of five cells per spot, which is comparable to the levels of 10× Visium. The results showed that Celloc achieved substantially higher $P_{\text{loc}}$ than other methods on both simulated cerebellum dataset and simulated hippocampus dataset (Figure 2e,f).

### 2.3 Celloc Reconstructs the Cell Spatial Pattern on Breast Cancer ST Data Across Different Reference ScRNA‐seq

In the above benchmarking on the simulation datasets, we evaluated the performance of Celloc on cerebellum and hippocampus datasets with varying noise levels and spot resolution, demonstrating its accuracy and robustness to expression noise. However, different reference scRNA‐seq data would lead to different mapping results due to the varying cell type definitions. Here, we tested the robustness of Celloc to different reference usages on breast cancer datasets. In total, four scRNA‐seq/ST combinations, encompassing two 10× Visium ST samples (ST sample1, ST sample2), including two scRNA‐seq datasets of HER2+ formalin‐fixed, paraffin‐embedded (FFPE) breast cancer specimen, and human ductal carcinoma in situ sample (DCIS1), were analyzed^[^ ^17^, ^25^ ^]^ (Experimental Section).

**Figure 3:**
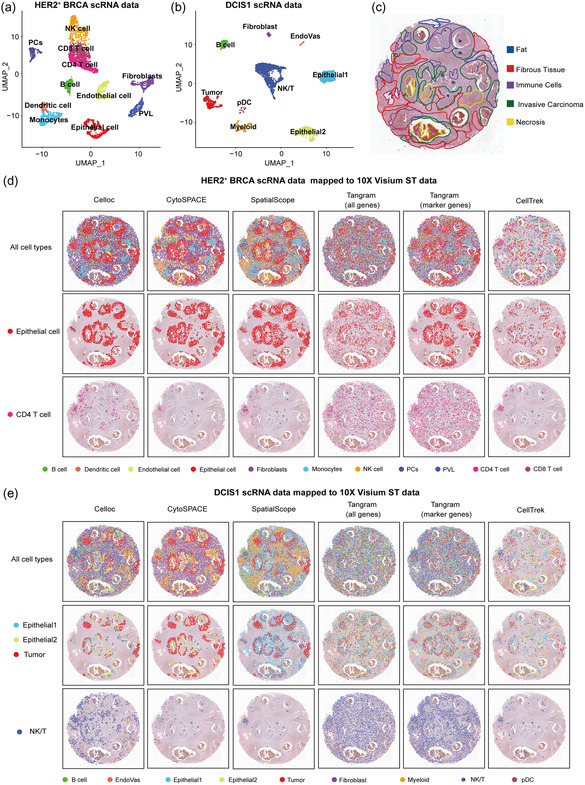
Visualization of (regular) mapping results of breast cancer ST dataset (sample1) with different scRNA‐seq references. To assess the robustness of methods toward different scRNA‐seq references, two alternative scRNA‐seq datasets, that is, HER2^+^ BRCA and DCIS1 scRNA‐seq datasets, were used as the reference mapped to the 10× Visium breast cancer ST sample1. a) UMAP of HER2^+^ BRCA scRNA‐seq data, showing 11 cell types, including epithelial cells but not tumor cells. b) UMAP of DCIS1 scRNA‐seq data, showing nine cell types including tumor cells and two types of epithelial cells. c) H&E image of the 10× Visium ST sample1 with five different labeled regions. d) Visualization of the mapping results by Celloc and the previous methods for all cell types, epithelial cells and CD4 T cells, using the HER2 + BRCA scRNA‐seq data and ST sample1 data. e) Visualization of the mapping results by Celloc and the previous methods for all cell types, epithelial 1/2 subgroups plus tumor cells and NK/T cells, using the DCIS1 scRNA‐seq data and ST sample1 data.

The HER2 + BRCA scRNA‐seq data with 3977 cells has 11 cell types including epithelial cells but not tumor cells (**Figure** **3**a). The DCIS1 scRNA‐seq data with 3587 cells has 9 cell types including tumor cells and two types of epithelial cells (Figure 3b). As for the ST sample1 data, five different regions have been labeled on histopathology image (Figure 3c). We assume by default that there are five cells in each spot on average. The actual number of cells mapped to each spot is shown in Figure S6, Supporting Information. We first applied Celloc to map the HER2 + BRCA scRNA‐seq data to ST sample1 data and compared it with CytoSPACE,^[^ ^15^ ^]^ SpatialScope,^[^ ^13^ ^]^ Tangram,^[^ ^16^ ^]^ and CellTrek.^[^ ^17^ ^]^ Celloc accurately reconstructed cellular spatial structures with distinct cell types located in different histological regions. CytoSPACE showed similar spatial patterns compared with Celloc, whereas Tangram and CellTrek could not reconstruct accurate spatial organizations (Figure 3d). We observed that epithelial cells were mapped to regions of invasive carcinoma, while CD4 T cells were mapped to regions of immune cells on histological image and areas surrounding invasive carcinoma. Similarly, we applied Celloc to map the DCIS1 scRNA‐seq data to ST sample1 data. Celloc accurately reconstructed the spatial structures of distinct cell types in ST sample1 data. CytoSPACE was affected by the results of deconvolution resulting in some cell types was missing. SpatialScope, Tangram, and CellTrek could not reconstruct accurate spatial organizations (Figure 3e). Epithelial subgroup 1 cells (Epithelial1), epithelial subgroup 2 cells (Epithelial2), and tumor cells were mapped to regions of invasive carcinoma, but each had a different focal area. NK/T cells were mapped to regions of immune cells on histological image and areas surrounding invasive carcinoma. In addition, we found that the mapping area of epithelial cells in the first scRNA‐seq/ST combinations highly overlapped with the mapping area of epithelial1, epithelial2, and tumor cells in the second scRNA‐seq/ST combinations, which was consistent with the conclusion from the previous research that most tumor cells in breast cancer originate from epithelial cell malignancies.^[^ ^26^ ^]^

Similarly, when applying Celloc to ST sample2 data, Celloc could also outperform other methods no matter using HER2 + BRCA scRNA‐seq or using DCIS1 scRNA‐seq data as the reference (Figure S7, Supporting Information). In both cases, epithelial cells (or their subgroups) and tumor cells were accurately mapped to the tumor area in the histopathological image and the mapping area highly overlapped. The actual number of cells mapped to each spot in ST sample2 data is shown in Figure S6, Supporting Information.

The above results demonstrate that Celloc is relatively more robust to the usage of scRNA‐seq reference. However, it should also be clarified that similar to other single‐cell‐to‐spot mapping methods, Celloc also requires scRNA‐seq data and ST data from the same region or tissue type to ensure the best performance.

### 2.4 Celloc Reveals Spatial Subclonal Heterogeneity of DCIS Breast Cancer

**Figure 4:**
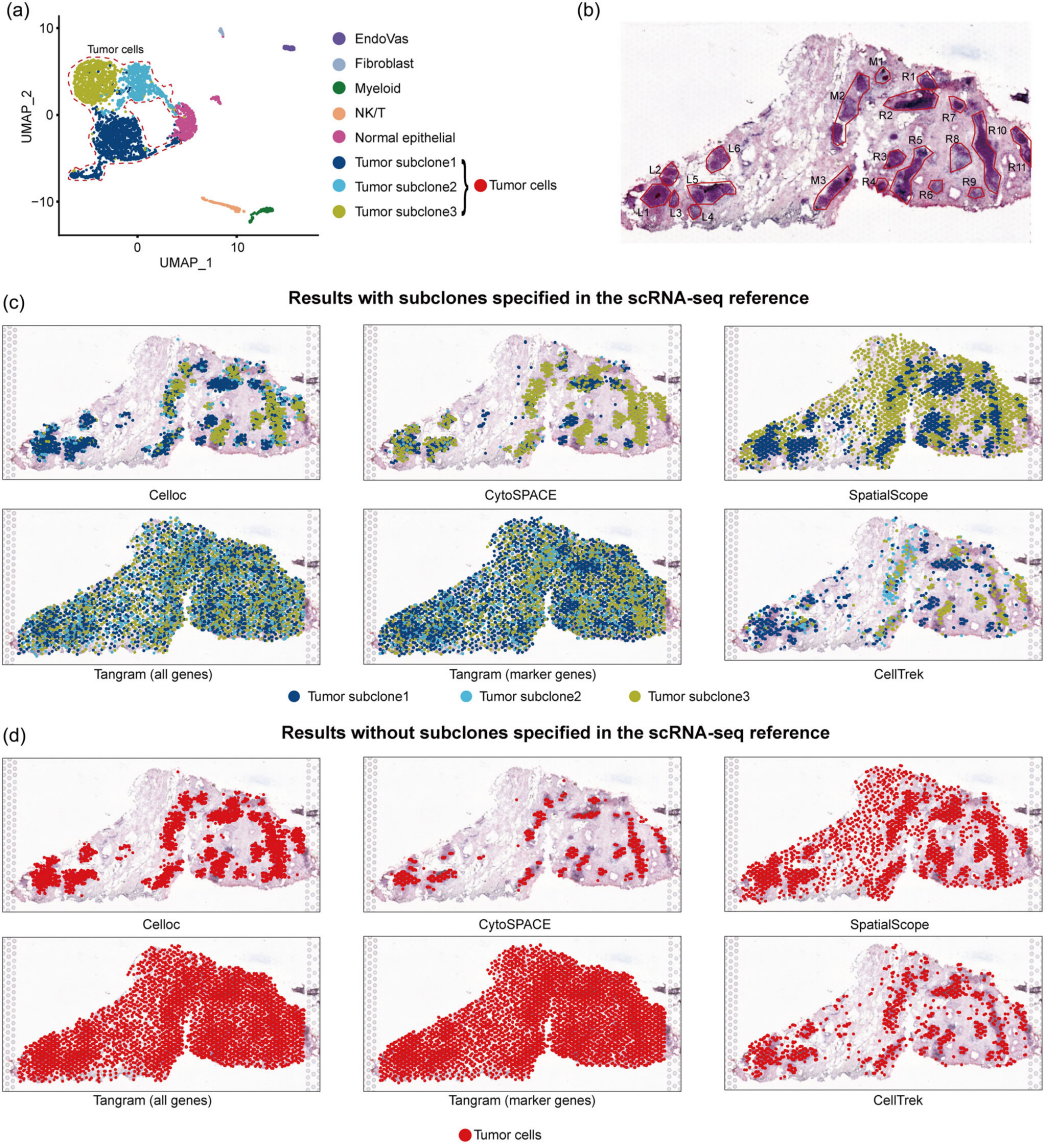
Visualization of (regular) mapping results of tumor subclones in DCIS dataset with and without subclone type specified in scRNA‐seq reference. The ability of Celloc and the previous methods to accurately recapitulate the spatial heterogeneity between different subclones, even without subclone annotation in the scRNA‐seq reference, was tested. a) UMAP of DCIS2 scRNA‐seq reference, showing the six (five nontumor cell types plus tumor cell) or the eight (five nontumor cell types plus three tumor subclones specified) cell type categorization. b) H&E image of the DCIS2 tissue section with annotated ductal tumor regions. c) Visualization of the mapping results of tumor subclones with subclones specified in the scRNA‐seq reference. d) Visualization of the mapping results of tumor cells without subclones specified in the scRNA‐seq reference. Notably, Celloc and CellTrek does not take cell type annotation in the scRNA‐seq reference into consideration; therefore, same results were obtained by Celloc and CellTrek using scRNA‐seq references with/without the subclone type annotation.

The definition of cell types, especially subtypes, could be ambiguous in real datasets. Tumor cells and their subclones constitute typical samples for such cases. Here, we tested the robustness of Celloc to different cell type annotations based on another DCIS dataset (DCIS2).^[^ ^17^ ^]^ The scRNA‐seq data with 2933 cells from the DCIS2 dataset has two different set cell type annotations: six cell types (five nontumor cell types plus tumor cells) and eight cell types (the tumor cells were categorized into three subclones, see Experimental Section for more details; **Figure** **4**a). As for the ST data from the DCIS2 dataset, 20 ductal tumor regions have been labeled on histopathology (Figure 4b).

To investigate the spatial distribution of three tumor subclones, we applied Celloc to the DCIS2 scRNA‐seq and ST data. The actual number of cells mapped to each spot in DCIS2 ST data is shown in Figure S6, Supporting Information. When using the eight cell types annotation with subclones specified in the scRNA‐seq reference, Celloc accurately reconstructed cellular spatial structures with distinct cell types located in different histological regions (Figure S8a, Supporting Information). We observed that most tumor subclones were located in the DCIS regions on the H&E slide (Figure 4c). Moreover, different tumor subclones exhibited distinct spatial patterns, reflecting extensive intratumor heterogeneity.^[^ ^27^ ^]^ Specifically, subclone2 was dispersed in many ductal areas; subclone3 was mainly concentrated on the right (R) ducts; subclone1 was presented in both left (L) and right (R) ductal regions but exhibited a well‐clustered distribution. CytoSPACE^[^ ^15^ ^]^ and CellTrek^[^ ^17^ ^]^ could also largely capture the spatial distribution of subclones but with less clear patterns. More specifically, CytoSPACE mapped the spatial location of subclone1 to a lesser extent in comparison with Celloc, the mapping of subclone3 was less obvious in CellTrek results, while SpatialScope^[^ ^13^ ^]^ and Tangram 16 did not produce reasonable subclone mapping results in this dataset.

For Celloc and CellTrek, the results would not be affected by cell type annotations since their calculation process did not involve cell type annotations. While for the other methods, the tumor and subclone regions were different when using the annotation without specified subclones, indicating that these methods would be affected by cell annotations (Figure 4c, and S8b, Supporting Information). These results indicated that Celloc could capture the spatial heterogeneity of cell types without being affected by the ambiguous cell type annotation in the input scRNA‐seq reference data.

### 2.5 Celloc Infers Spatial Tumor‐Immune Microenvironment of a DCIS Tissue

**Figure 5:**
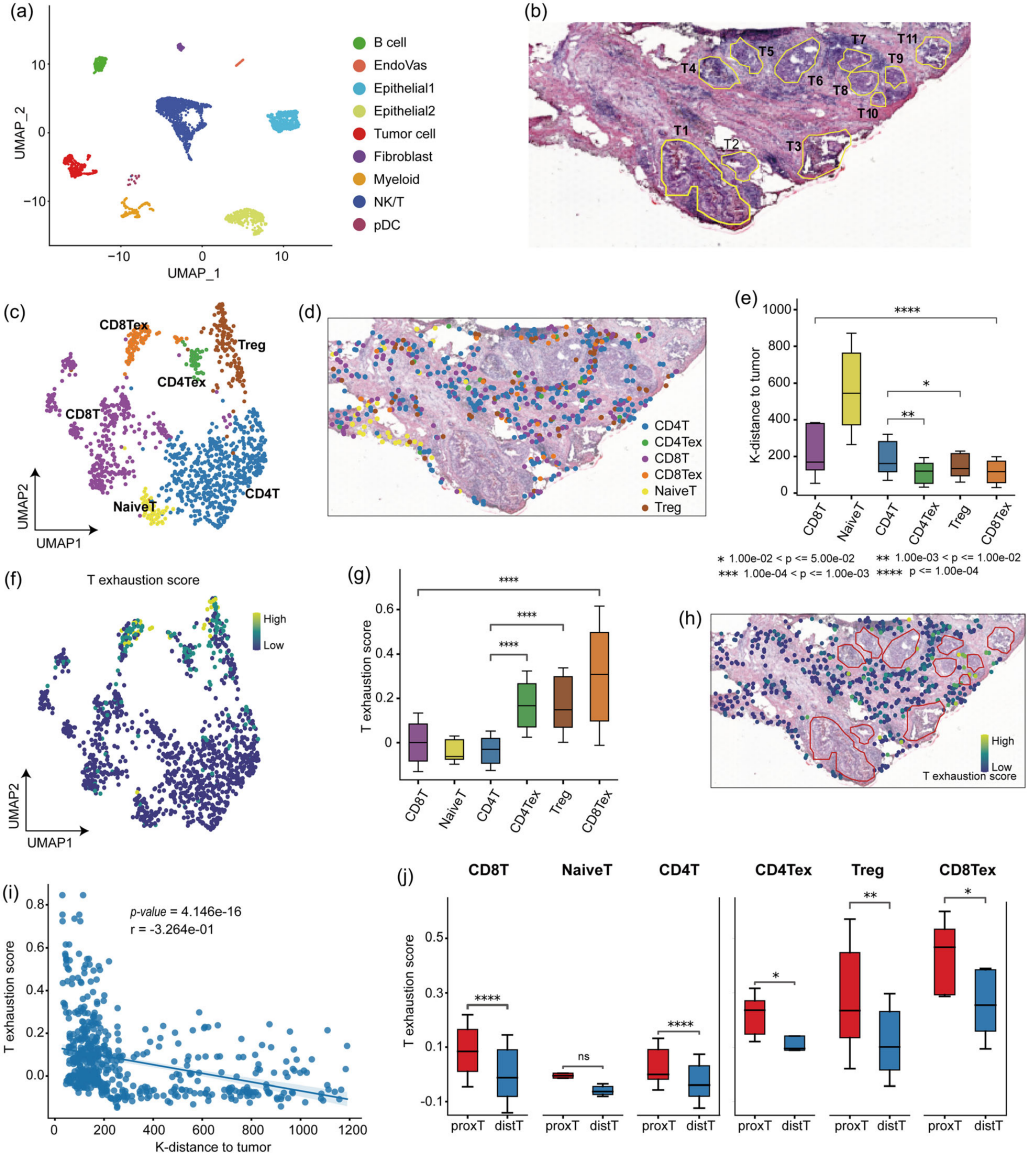
Spatial tumor‐immune microenvironment analysis based on the Celloc mapping result. a) UMAP of DCIS1 scRNA‐seq reference, showing nine cell types, including two epithelial cell subgroups, tumor cells, endothelial cells, fibroblasts, myeloid cells, NK/T cells, B cells, and plasmacytoid dendritic (pDC) cells. b) H&E image of the DCIS1 tissue section with annotated ductal tumor regions. c) UMAP of six T cell states, including the Naive T (NaiveT), CD4 + T (CD4T), CD8 + T (CD8T), regulatory T cells (Treg), exhausted CD4 + T (CD4Tex), and exhausted CD8 + T (CD8Tex). d) The spatial distribution of T cells in the six cell states in Celloc regular mapping results. e) The boxplots comparing the proximity of different state T cells to the tumor cells (assessed by K‐distance approach to their five nearest tumor cells based on Euclidean distance). Statistical significance is evaluated by Wilcoxon rank‐sum test. The exact *P*‐values (up to bottom) are 6.418e‐05, 1.092e‐02, 2.023e‐03. f) UMAP showing T exhaustion scores of the six T cell states. g) The boxplots comparing T exhaustion scores of the six T cell states. The exact *P*‐values (up to bottom) are 1.563e‐22, 2.154e‐34, 6.446e‐13. h) The spatial distribution of T cells with different T exhaustion scores. i) The Pearson correlation between K‐distances and T exhaustion scores. j) The boxplots comparing T exhaustion scores of tumor‐distal (distT) and tumor‐proximal (proxT) groups in the mapping result. The exact *P‐*values (left to right) are 3.161e‐06, 1.283e‐01, 8.016e‐08, 3.049e‐02, 1.123e‐03, 3.346e‐02.

To check whether Celloc could more accurately depict the tumor‐immune microenvironment interactions, we applied Celloc to DCIS1 scRNA‐seq and ST data with synchronous invasive components from CellTrek.^[^ ^17^ ^]^ The actual number of cells mapped to each spot in DCIS1 ST data is shown in Figure S6, Supporting Information. For the scRNA‐seq data, there were nine cell types, including two epithelial, tumor cell, endothelial, fibroblasts, myeloid, NK/T, B, and plasmacytoid dendritic cells (pDC) (**Figure** **5**a). Histopathological analysis of the H&E image identified 11 ductal regions with tumor cells (T1–T11) and intervening areas that contained stromal and immune cells (Figure 5b). Using Celloc, we reconstructed cellular spatial structures with distinct cell types located in different histological regions (Figure S9, Supporting Information). Tumor cells were mapped to the histologically defined DCIS regions and NK/T cells were mapped to areas surrounding ducts and stromal regions. CytoSPACE^[^ ^15^ ^]^ could also largely capture the spatial pattern with lower accuracy, while the other three methods did not produce an accurate and comprehensive cell type spatial distribution pattern (Figure S9a, Supporting Information). The difference is more obvious for tumor cells (Figure S9b, Supporting Information) and NK/T cells (Figure S9c, Supporting Information), where Tangram captured less tumor region but overestimated NK/T distribution, while SpatialScope was exactly the opposite. CellTrek resulted in a highly discrete mapping without a clustered topological domain in the spatial data.

From Celloc mapping results, we noted that some T cells were proximal to the tumor regions while some others were distal. Similar to CellTrek 17, we further grouped T cells into six cell states, including the Naive T (NaiveT), CD4 + T (CD4T), CD8 + T (CD8T), regulatory T cells (Treg), exhausted CD4 + T (CD4Tex), and exhausted CD8 + T (CD8Tex) (Figure 5c). We analyzed the spatial mapping distribution of T cell states on the H&E image. Notably, the Tregs, CD4Tex, and CD8Tex cells were mostly proximal to the tumor cells (Figure 5d), which was verified by the K‐distances of the T cells to their five nearest tumor cells based on Euclidean distance (Figure 5e). We calculated T exhaustion scores and found that the immunosuppressive T cells (Treg, CD4Te, and CD8Te) had higher exhaustion scores compared with the nonsuppressive T cells (Figure 5f,g), which is consistent with previous finding.^[^ ^28^ ^]^ We further found that the K‐distances of the T cells showed a negative correlation trend to the T exhaustion scores: in other words, the closer the T cells were to the tumor area, the higher their exhaustion score (Figure 5h,i). Finally, we divided T cells into tumor‐distal (distT) and tumor‐proximal (proxT) groups based on their median k‐distance and found that the proxT group showed significantly higher exhaustion score than the distT group (Figure 5j), in line with the presence of immunosuppressive microenvironment near the DCIS ductal regions. These results confirmed Celloc's ability to infer the spatial pattern in tumor immune microenvironment.

### 2.6 Celloc Signifies the Spatial Distribution and Gene Spatial Expression Patterns of Specific Cell Types in Myocardial Infarction (MI)

We further applied Celloc to map the mouse myocardial infarction (MI) scRNA‐seq data^[^ ^29^ ^]^ with 4486 cells which contains five cell types to the mouse MI ST data.^[^ ^30^ ^]^ The spatial mapping results showed that the number of cardiomyocytes in the infarct area was significantly reduced, while the number of fibroblasts and macrophages in the infarction fibrotic area was significantly increased, in line with the known fibrosis and immune cell infiltration processes during MI^[^ ^29^ ^]^ (Figure S10a, Supporting Information). The actual number of cells mapped to each spot in MI ST data is shown in Figure S6, Supporting Information.

We further tried to compare the expression pattern between cardiomyocytes located proximal and distal to the fibrosis region. However, we noted that regular single cell‐to‐spot mapping is not suitable for this case: there were only 997 cardiomyocytes in the scRNA‐seq data, while 6790 cardiomyocytes were mapped in the ST data, meaning that the vast majority of cardiomyocytes mapped to the ST data were duplicated from 997 cardiomyocytes that shared the same expression profile, which could result in a non‐negligible bias. To this end, we further exploited Celloc's greedy mapping function to predict the most likely location of each cell in the tissue section. The results showed that, although fewer cells could be mapped without duplication of cells in scRNA‐seq data, obvious enrichment of fibroblasts and macrophages was found in the fibrosis region (Figure S10b, Supporting Information). The spatial topology also became more precise with greedy mapping, as the fibroblasts and macrophages are clustered at the rim but not the core of fibrotic area, in line with their direct profibrosis role to progressively expand the fibrotic area during MI. More interestingly, the mapped cardiomyocytes could be organized into two groups according to their spatial distance to the infarction fibrotic area, enabling a more unambiguous comparison between the two groups (Figure S10b, Supporting Information). We used the mapped fibroblasts as the proxy to define the fibrotic area and calculated K‐distances of the cardiomyocytes to their five nearest fibroblasts based on Euclidean distance. The top 30% nearest cardiomyocytes were defined as the proximal cardiomyocytes group (Prox_CM), and the top 30% farthest ones were defined as distal cardio myocytes group (Dist_CM). Since almost all fibroblasts were distributed in the infarct area (Figure S10b, Supporting Information), Prox_CM and Dist_CM also represented cardiomyocytes near and far from the infarction area, respectively.

By comparing gene expression profiles of Prox_CM versus Dist_CM, we identified 63 upregulated genes and 11 downregulated genes (fold change > 1.5, adjust *P*‐value < 0.05) in Prox_CM (Figure S10c, Supporting Information). Some of these differentially expressed genes have been reported for their important roles in MI. For example, upregulated gene Flna mediates the progression of MI and atherosclerosis,^[^ ^31^ ^]^ and Kcnq1ot1 regulates cardiomyocyte autophagy and apoptosis both in vivo and in vitro.^[^ ^32^ ^]^ Among the downregulated genes, the lncRNA Mhrt is a protective factor for cardiomyocyte and the plasma concentration of Mhrt may serve as a biomarker for MI diagnosis in humans’ acute MI.^[^ ^33^ ^]^ GSVA analysis of gene ontology functional terms revealed that cardiomyocytes in different spatial locations had different functions. We found that Prox_CM group upregulated some development‐related pathways (Figure S10d, Supporting Information). These results demonstrated that Celloc's regular and greedy mapping can produce reasonable and complementary observations, making Celloc more flexible in downstream task applications.

## 3 Discussion

In this study, we introduce Celloc, a deep learning approach that maps scRNA‐seq data to ST data with high resolution and predicts the spatial location of each cell. We demonstrate Celloc's superior performance on simulated ST data, surpassing other methods in both regular and greedy single cell‐to‐spot mapping tasks. In addition, using regular mapping, Celloc could also estimate the cell type proportion of each spot‐like traditional deconvolution methods. The results showed that Celloc outperformed the state‐of‐the‐art deconvolution methods. We further apply Celloc to various real‐world datasets: breast cancer ST data across different scRNA‐seq references, DCIS breast cancer revealing spatial subclonal heterogeneity, DCIS tissue inferring spatial tumor‐immune microenvironment, and MI analyzing spatial distribution and gene expression patterns of specific cell types. Our results illustrate Celloc's accurate reconstruction of cell spatial patterns and its superior sensitivity‐specificity balance and robustness to background noise, spatial resolution variations, scRNA‐seq/ST combinations, and alterations in cell type annotations compared to existing methods.

Celloc's superior performance stems from its comprehensive objective function, incorporating gene expression similarity, embedding correlation, and cell quantity per spot (Figure 1). This approach differs from Tangram, which emphasizes cell density and gene expression. In Celloc, the learnable mapping matrix is initialized based on the estimated cell numbers per spot and dynamically adjusted during optimization. In contrast, Tangram initializes its mapping matrix with a normal distribution, potentially limiting the search space. CytoSPACE showed competitive performance in our benchmarking tests (Figure 2, 3, 4, and S2–S4, Supporting Information). CytoSPACE and Celloc leverage similar methods for estimating expected cell numbers per spot. Nonetheless, Celloc futher employs a weighted cell quantity term to adjust the intensity of cell number constraints (Figure 1). In contrast, CytoSPACE adheres more strictly to estimated cell quantities, mapping all sampled cells to ST data even if not necessary, resulting in reduced accuracy when scRNA‐seq and ST data have vastly different cell counts. Additionally, CytoSPACE's performance depends heavily on ST deconvolution results, while current Celloc architecture is deconvolution process independent. CellTrek utilizes random forest modeling for spatial coordinate prediction but lacks single‐cell resolution cell composition of each spot in the ST data. Consequently, it tends to be conservative in mapping cells to spots, resulting in less resolved spatial topology (e.g., Figure 3, 4 and S8 and S9, Supporting Information). SpatialScope counts cells per spot and identifies cell types to calculate cell numbers per type per spot, but does not generate exact single cell‐to‐spot mappings. Thus, we compared Celloc with SpatialScope based on cell type assignment error rates per spot, as direct mapping accuracy comparison was not feasible.

Moreover, neither CytoSPACE, SpatialScope, nor CellTrek supports greedy single cell‐to‐spot mapping. When the number of each cell type in scRNA‐seq data and ST data is imbalanced, arbitrary over‐ or downsampling of the under‐ or over‐represented cell types may result in an inaccurate and biased location assignment for each cell in the scRNA‐seq data. In Celloc, the greedy mapping function that maps every single cell to one best‐matched spot is also enabled, without substantial modification of the algorithm (Figure S1, Supporting Information). Our benchmarking results suggest the better performance of Celloc in greedy mapping (Figure 2e,f). NovoSpaRc greedy mapping did not obtain satisfactory results. An important reason is that NovoSpaRc does not use the expression profile of ST data, but only uses the spatial location information as candidate coordinates of cells.

On the other hand, Celloc also has several limitaions. First, Celloc requires scRNA‐seq data and ST data from the same region or tissue type to ensure the best performance. In addition, Celloc's use of scRNA‐seq data and ST data with similar cell numbers will be more conducive to downstream analysis. On regular mapping, when the number of cells in scRNA‐seq is much smaller than the expected cell number in ST data, many cells will be duplicated and mapped to multiple spots, which is not conducive to downstream analysis based on expression profiles, and greedy mapping would be an important step for such cases. Furthermore, Celloc does not utilize the image texture features of tissue sections in the mapping algorithm. With the popularity of paired high‐resolution H&E images and ST data, establishing relationships between histopathological images and expression profiles may improve mapping accuracy.

We also noted that although the number of mapped cells would vary between the regular and greedy mapping results, the overall distributions of cell type can significantly overlap (Figure S10, Supporting Information). Therefore, users can choose different mapping methods based on different downstream analysis tasks. In all, we hope that our proposed Celloc will help advance joint analysis of scRNA‐seq data and ST data and create a new avenue for future exploration of the spatial organization of cells within complex tissues.

## 4 Conclusion

Celloc is a GATE and multiple loss‐based method allows either dissecting cell composition of each spot (regular mapping) or predicting spatial location of every cell in scRNA‐seq data (greedy mapping). Celloc enables flexible single cell‐to‐spot mapping, which can 1) fill ST spots with a suitable number of cells from scRNA‐seq to enhance the quality of ST data in terms of resolution and gene expression quantity (regular mapping) and 2) assign the spatial location for every cell in scRNA‐seq data to completely investigate the spatial pattern across the full scRNA‐seq dataset (greedy mapping). On both simulated and real ST datasets, we found that Celloc substantially outperformed the related methods for resolving single cell spatial distribution. In all, we hope that our proposed Celloc will help advance joint analysis of scRNA‐seq data and ST data and create a new avenue for future exploration of the spatial organization of cells within complex tissues.

## 5 Experimental Section

5.1

5.1.1

##### Celloc Data Preparation Pipeline

The input of Celloc was ST data and scRNA‐seq data from the same tissue or region. We used the real number (**R**) matrix $C=R^{m\times g}$ and $S=R^{w\times g}$ for single‐cell and ST gene expression profiles with *m* cells, *w* spots, and *g* genes. The data preparation had two steps. This makes the expression data from scRNA‐seq and ST more comparable.

In the first step, we preprocessed the scRNA‐seq and ST data to get the common genes and normalized the expression profiles. We used the scanpy package^[^ ^34^ ^]^ to analyze all scRNA‐seq and ST datasets. We removed genes in no more than 1 cell or 1 spot. For both expression profile matrices in scRNA‐seq and ST, we normalized the values to counts per million and log2 transformed them using the sc.pp.normalize_total() and sc.pp.log1p() functions from scanpy.

In the second step, we estimated the number of cells $N=[n_{1},n_{2},\ldots ,n_{w}]$ in ST spots based on RNA or H&E image. That is to say, we estimated that $n_{j}$ cells should be mapped to the *j*‐th spot in ST data. The basic assumption is that gene expression in a cell is related to mRNA amount, measured by unique molecular identifiers (UMIs) per cell.^[^ ^35^ ^]^ Following CytoSPACE,^[^ ^15^ ^]^ the sum of UMIs per ST spot was assumed to be a reasonable proxy for the number of cells per spot. We normalized UMIs to counts per million per spot and log2 transformed them. We fit a linear relation between two anchor spots to estimate cells per ST spot. The first anchor spot had the minimum nonzero normalized UMIs and one cell. The second anchor spot had the median‐normalized UMIs and the mean cells per spot. We used five as the default mean cells per spot for 10× Visium samples, but users can change it. Segmentation of nuclei in H&E images is more accurate to count cells per spot. We used StarDist^[^ ^21^ ^]^ for nucleus segmentation on H&E images.

##### Graph Attention Autoencoder

GATE^[^ ^20^ ^]^ is a deep learning method that can learn low‐dimensional latent representations from high‐dimensional data. Celloc learnt low‐dimensional latent embeddings via GATE on scRNA‐seq data and ST data, respectively, while retaining the topological structure and content information. The GATE consisted of two parts: encoder and decoder.

The encoder in our architecture took the normalized gene expressions and adjacency matrix as input and generated the latent embedding by collectively aggregating information from its neighbors. For scRNA‐seq data, the k‐nearest‐neighbor algorithm was used to find the k‐nearest data nodes to a given query nodes based on gene expression, while the adjacency matrix for ST data was based on spatial distance.

Each encoder layer generated new representations of nodes utilizing their neighbors’ representations according to their relevance. To adaptively learn the similarity between neighboring spots, we employed a self‐attention mechanism that was widely used for graph neural networks. In the k‐th encoder layer, the edge weight from node *i* to its neighbor node *j* was computed as follows.

(1) $$r_{ij}^{\left(k\right)}=\text{Sigmoid}(v_{s}^{{\left(k\right)}^{T}}\sigma (W^{\left(k\right)}h_{i}^{\left(k-1\right)})+v_{t}^{{\left(k\right)}^{T}}\sigma (W^{\left(k\right)}h_{j}^{\left(k-1\right)}))$$

where $W^{\left(k\right)}$, $v_{s}^{\left(k\right)}$, $v_{t}^{\left(k\right)}$ are the trainable parameters of the *k*‐th encoder layer, *σ* denotes the activation function, and sigmoid represents the sigmoid activation function. To make the relevance coefficients comparable, we normalized them using the softmax function as follows.

(2) $$\alpha_{ij}^{\left(k\right)}=\frac{exp(r_{ij}^{\left(k\right)})}{\sum_{l\in A_{i}}exp(r_{il}^{\left(k\right)})}$$

where $A_{i}$ represents the neighborhood of node *i* (i.e., a set of nodes connected to node *i* according to the adjacency matrix, including node *i* itself).

By considering expression profiles as initial node representations (i.e., $h_{i}^{\left(0\right)}=x_{i},\forall i\in \{1,2,\ldots ,m/w\}$), the k‐th encoder layer generates the representation of node *i* in layer *k* as follows.

(3) $$h_{i}^{\left(k\right)}=\sum_{j\in A_{i}}\alpha_{ij}^{\left(k\right)}\sigma (W^{\left(k\right)}h_{j}^{\left(k-1\right)})$$

After applying *L* encoder layers, we considered the output of the last layer as the final node embedding ***Z***.

The decoder reverses the latent embedding back into a reconstructed normalized expression profile. The calculation of normalized correlation was the same as that of the encoder, represented by ${\hat{\alpha }}_{ij}^{\left(k\right)}$. By treating the output of the encoder as the input of the decoder (i.e., ${\hat{h}}_{i}^{\left(L\right)}=h_{i}^{\left(L\right)}$), the *k*‐th decoder layer reconstructs the representation of node *i* in layer *k*‐1 as follows.

(4) $${\hat{h}}_{i}^{\left(k-1\right)}=\sum_{j\in A_{i}}{\hat{\alpha }}_{ij}^{\left(k\right)}\sigma ({\hat{W}}^{\left(k\right)}{\hat{h}}_{j}^{\left(k\right)})$$

After applying *L* decoder layers, we considered the output of the last layer as the reconstructed normalized expressions (i.e., ${\hat{x}}_{i}={\hat{h}}_{i}^{\left(0\right)}$). Finally, the objective of GATE was to minimize the reconstruction loss of normalized expressions as follows.

(5) $$\sum_{i=1}^{\text{nodes}}∥(x_{i}-{\hat{x}}_{i}){∥}_{2}$$

where nodes represent the number of cells in scRNA‐seq data or spots in ST data.

##### Celloc Regular Single Cell‐to‐Spot Mapping Algorithm

Celloc leverages deep learning optimization^[^ ^36^ ^]^ to formulate the assignment problem that aims at mapping single cells in scRNA‐seq data to the spots in ST data. The output of the mapping algorithm is the learnt mapping matrix ***M***. The matrix value represents the mapping score of the cell and spot. According to the estimated number *n* of cells in each spot, cells with the top *n* highest mapping score were mapped to this spot.

We first initialized a matrix $M=R^{m\times w}$ as the mapping between the scRNA‐seq data and ST data provided $\sum_{j}^{w}M_{ij}=1$. During the training process, we ensured that $0\leq M_{ij}\leq 1$ and $\sum_{j}^{w}M_{ij}=1$ by applying the softmax. To learn the mapping matrix, we minimized the following objective function with respect to ***M***.

(6) $$L=\sum_{i,j}\text{KL}(C_{i,*},S_{j,*})M_{ij}+\sum_{i,j}D_{ij}M_{ij}+\lambda \frac{1}{w}\sum_{j=1}^{w}\left|N_{j}-\sum_{i}^{m}M_{ij}\right|$$

where KL indicates the Kullback–Leibler divergence and * indicates matrix slicing. The first term is the expression‐based mapping term: it requires that the gene expression of the mapped cells and spots should be as similar as possible and ensure that the cells and spots with smaller KL divergence have higher mapping scores. The second term is the expression‐based mapping term: it ensures that mapped cells and spots have high PCC in the embedding space. Instead of KL divergence, we used the Pearson distance matrix $D=R^{m\times w}$ to represent the similarity in the embedding space, where $D_{\phi \psi }=1-PCC(Z_{\phi ,*}^{\left(sc\right)},Z_{\psi ,*}^{\left(st\right)})$ is the Pearson distance between cell *ϕ* and spot *ψ*. The third term is the cell quantity constraint term: it requires that the number of cells mapping to each spot is equal to the expected cell number per spot. This term also increases the variability of mapping scores. The parameter *λ* is the weight for the cell quantity constraint terms in the loss function. The parameter was preliminarily optimized as 0.1 based on the performance on simulated data (Figure S11a, Supporting Information). In addition, we also performed an ablation study to verify the contribution of each loss term and found that all of the three terms are important to the superior performance of Celloc (Figure S11b, Supporting Information).

Based on the solved mapping matrix ***M***, a single cell‐to‐spot mapping task can be accomplished. Note that in some cases of this task, one cell would be mapped to multiple spots; at the same time, some other cells will not be considered.

##### Celloc Greedy Mapping Algorithm

The mapping matrix ***M*** generated by Celloc facilitates greedy single cell‐to‐spot mapping, wherein each cell in scRNA‐seq data was assigned to the spot with the highest mapping score. Figure S1, Supporting Information, elucidates the differing objectives of these tasks.

Despite the disparate aims, Celloc employs consistent data preprocessing and model training methodologies for both mapping tasks. The term weight parameter *λ* was optimized at 0.1 (Figure S11a, Supporting Information). Notably, even for the greedy mapping task, the cell quantity constraint term remained useful, effectively preventing erroneous cell aggregation into a minority of spots (Figure 1).

##### Using Slide‐Seq Datasets to Simulate ST Datasets

Slide‐seq datasets^[^ ^5^ ^]^ contain gene expression profiles, spatial locations, and cell‐type annotations of individual cells. We assessed Celloc's performance on regular and greedy mapping tasks using simulated Slide‐seq datasets derived from mouse cerebellum and hippocampus sections by CytoSPACE.^[^ ^15^ ^]^ To simulate ST data with different spatial resolutions, we adjusted the bin size to achieve average cell counts of 5, 15, and 30 cells per spot. To mimic technical variability across platforms, we introduced noise to the scRNA‐seq data. The noise level was controlled by the percentage of genes (pt) to perturb, with perturbed genes randomly selected from each cell and noise added from an exponentiated Gaussian distribution $2^{N(0,1)}$. We experimented with noise perturbation levels of 5%, 10%, and 25%.

##### Acquisition and Analysis of Human Ductal Carcinoma In Situ (DCIS) Data

The DCIS scRNA‐seq datasets and ST datasets were obtained from CellTrek.^[^ ^17^ ^]^ In the DCIS1 scRNA‐seq data, nine main cell types were annotated, including various epithelial cells, tumor cells, B cells, endovas, fibroblasts, myeloid cells, NK/T cells, and pDC. Subtypes of NK/T cells were further delineated through differential expression analysis and canonical marker expression. T cell exhaustion was characterized using a gene signature including PDCD1, CTLA4, LAG3, HAVCR2, CD244, CD160, and TIGIT.^[^ ^17^ ^]^

In the DCIS2 scRNA‐seq data, six main cell types were annotated: normal epithelial cells, tumor cells, endothelial cells, fibroblast cells, myeloid cells, and NK/T cells. Based on copy number profiles, three primary tumor subclones (subclone1–3) were identified across all tumor cells. For ST data, we selected spots covering tumor areas based on matched histopathology images to assess Celloc's accuracy in mapping tumors (or tumor subclones) to DCIS regions, irrespective of specified tumor subclones.

##### Collection and Analysis of ScRNA‐seq and ST Dataset of Breast Cancer

The HER2^+^ FFPE breast cancer scRNA‐seq data was obtained from a previous study.^[^ ^25^ ^]^ Eleven main cell types were annotated in the scRNA‐seq data, including B cells, dendritic cells, endothelial cells, epithelial cells, fibroblasts, monocytes, NK/T cells, PCs, PVL, CD4 T cells, and CD8 T cells. Two 10× Visium ST samples (ST sample1, ST sample2) were downloaded from the 10× Genomics official site. We tested the robustness of Celloc when using different reference scRNA‐seq data on the breast cancer datasets. In total, four scRNA‐seq/ST combinations were analyzed: HER2+ scRNA‐seq data and ST sample1; HER2^+^ scRNA‐seq data and ST sample2; DCIS1 scRNA‐seq data and ST sample1; DCIS1 scRNA‐seq data and ST sample2.

##### Collection and Analysis of ScRNA‐seq and ST Dataset of Myocardial Infarction (MI)

The MI scRNA‐seq data was obtained from a previous study.^[^ ^29^ ^]^ This dataset covers cardiomyocytes, endothelial cells, fibroblasts, smooth muscle cells, and macrophages from cardiac tissue in MI mouse models. For the ST data of mouse MI, we downloaded ST data of 7 days after MI from the GEO database.^[^ ^30^ ^]^

##### Performance Assessment Metrics

We used the following three metrics to evaluate the performance of Celloc for simulated data on the two tasks of single cell‐to‐spot mapping.

1) $P_{\text{map}}$: Similar to CytoSPACE,^[^ ^15^ ^]^ to assess the performance of regular single cell‐to‐spot mapping, locations that exactly matched the ground truth spots were counted as the correct assignments. The single‐cell mapping precision ($P_{\text{map}}$) for each cell type is

(7) $$P_{\text{map}}=\frac{N_{T}}{N_{\text{map}}}$$

where $N_{T}$ denotes the number of correct assignments for one cell type and $N_{\text{map}}$ denotes the number of all mapped cells with known ground truth locations for the same cell type. Therefore, $P_{\text{map}}$ is the fraction of cells per cell type mapped to correct ST spot.

2) Error rate: To determine the accuracy of cell type identification for each spot, we calculated the misclassification error rate, which represents the proportion of cells with misclassified cell type labels. To facilitate calculation, in simulation study, we assumed that we know the ground truth cell number in each spot. The error rate can be defined as

(8) $$\text{error rate}=\frac{N_{\text{error}}}{N_{\text{cell}}}$$

where $N_{\text{error}}$ denotes the number of misclassified cells and $N_{\text{cell}}$ denotes the total number of single cells in ST data.

3) $P_{\text{loc}}$: To evaluate the performance of Celloc in the greedy single cell‐to‐spot mapping, we calculated the precision of mapping across different cell types. Similar to $P_{\text{map}}$, the location prediction precision ($P_{\text{loc}}$) can also be defined in cell type‐wise manner as

(9) $$P_{\text{loc}}=\frac{N_{t}}{N_{\text{ct}}}$$

where $N_{t}$ denotes the number of correctly located cells for one cell type and $N_{\text{ct}}$ denotes the total number of cells for the same cell type.

##### Statistical Analysis

The statistical analysis was performed using the two‐tailed Wilcoxon test (*α* = 0.05). The methods for deep learning and bioinformatics analyses were stated in the corresponding subsections of Experimental Section.

## Code Availability

The Celloc code is available at GitHub: https://github.com/wwYinYin/Celloc.

## Conflict of Interest

The authors declare no conflict of interest.

## Supporting information

Supplementary Material

## Data Availability

The publicly available expression datasets analyzed in this work are available in previous studies and available at Figshare (https://figshare.com/articles/dataset/Celloc_data/25894927/1). The simulated mouse cerebellum and hippocampus can be obtained from https://github.com/digitalcytometry/cytospace. The HER2+ breast cancer scRNA‐seq data is available from the Gene Expression Omnibus (GEO) with accession numbers GSE176078. Two 10X Visium ST samples (ST sample1, ST sample2) are obtained from https://www.10xgenomics.com/resources/datasets. The DCIS dataset is available in the previous reference PMID: 35314812. The scRNA‐seq data of myocardial infarction is available from the GEO with accession numbers GSE129175. The ST data of myocardial infarction is available from the GEO with accession numbers GSE165857.
