# Supplementary material for: Accurate and Flexible Single Cell to Spatial Transcriptome Mapping with Celloc
Source: Small Sci. 2024 Jun 26;4(10):2400139. doi: 10.1002/smsc.202400139 (PMC11934999; doi:10.1002/smsc.202400139)
Supplement: Supplementary file 1 — Supplementary Material [file SMSC-4-2400139-s001.pdf]

Supplementary Figures

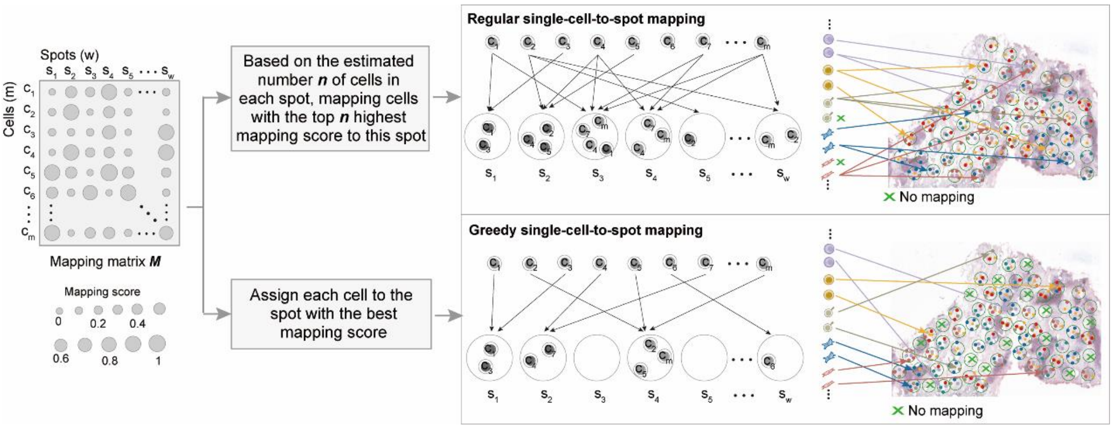

Supplementary Fig. 1. Schema comparing Celloc's regular and greedy single-cell-to-spot mapping tasks. For regular single-cell-to-spot mapping, based on the estimated number *n* of cells in each spot, Celloc mapped cells with the top *n* highest mapping score to this spot. The regular mapping aimed to fill ST spots with suitable number of cells from scRNA-seq to enhance the quality of ST data in terms of resolution and gene expression quantity. For greedy single-cell-to-spot mapping, Celloc assigned each cell to the spot with the best mapping score. The greedy mapping aimed to assign the spatial location for every cell in scRNA-seq data to completely investigate the spatial pattern across the full scRNA-seq dataset.

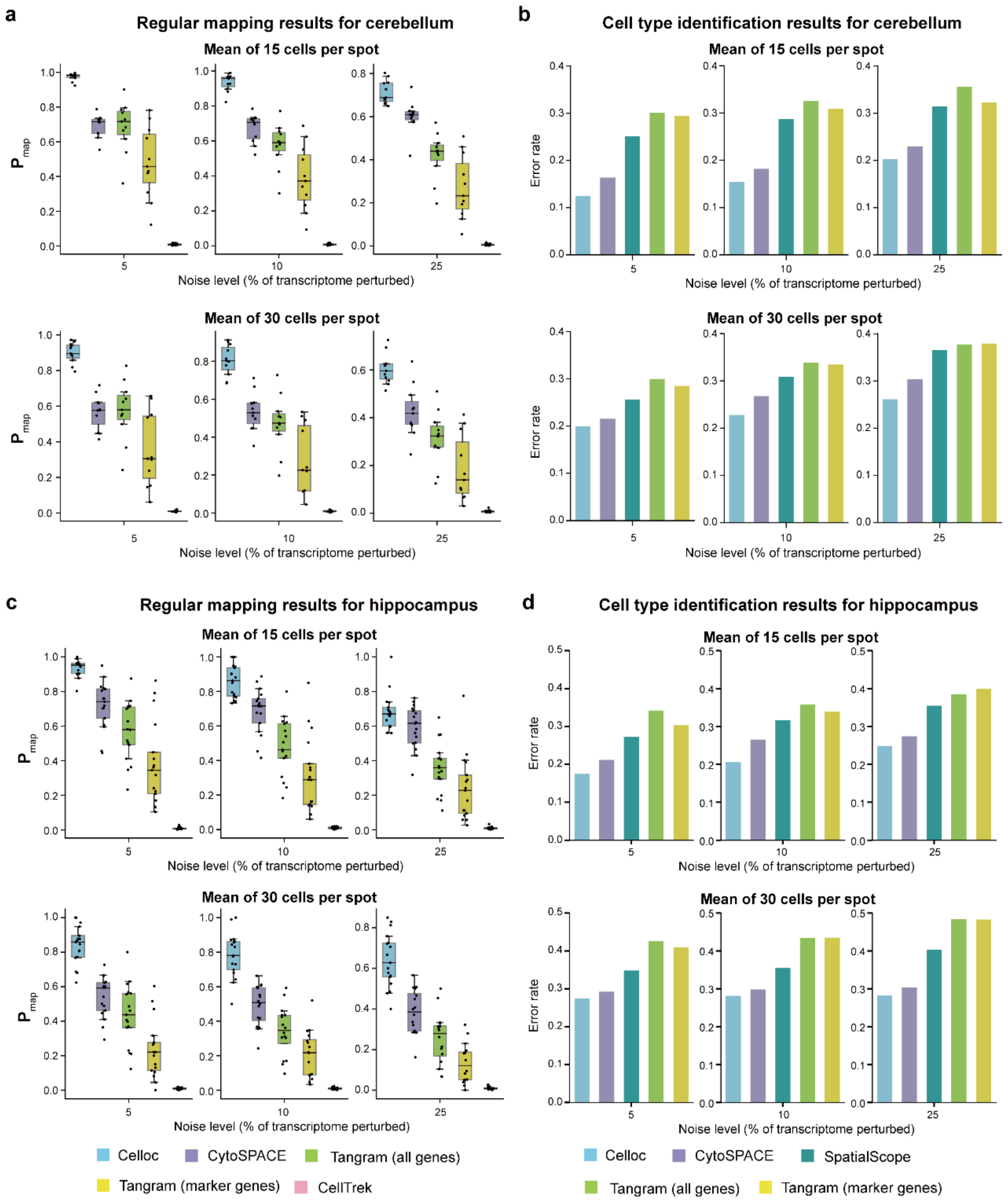

Supplementary Fig. 2. Evaluation of Celloc's performance on simulated cerebellum and hippocampus datasets. The simulated ST data with mean of 15 cells and 30 cells per spot. **(a)** On the simulated cerebellum datasets, the performances of Celloc and the previous methods for regular mapping across different noise levels were compared. Each point represents a single cell type (mouse cerebellum, n = 11). Boxplots: center line, median; box limits, upper and lower quartiles. The boxplots comparing the mapping precisions (P_{map}). **(b)** The barplots comparing cell type identification accuracy by calculating the cell type misclassification *error rate* on the simulated cerebellum datasets. **(c)** The performances of Celloc and the previous methods for fregular mapping on the simulated hippocampus datasets. **(d)** The barplots comparing cell type identification accuracy on the simulated hippocampus datasets.

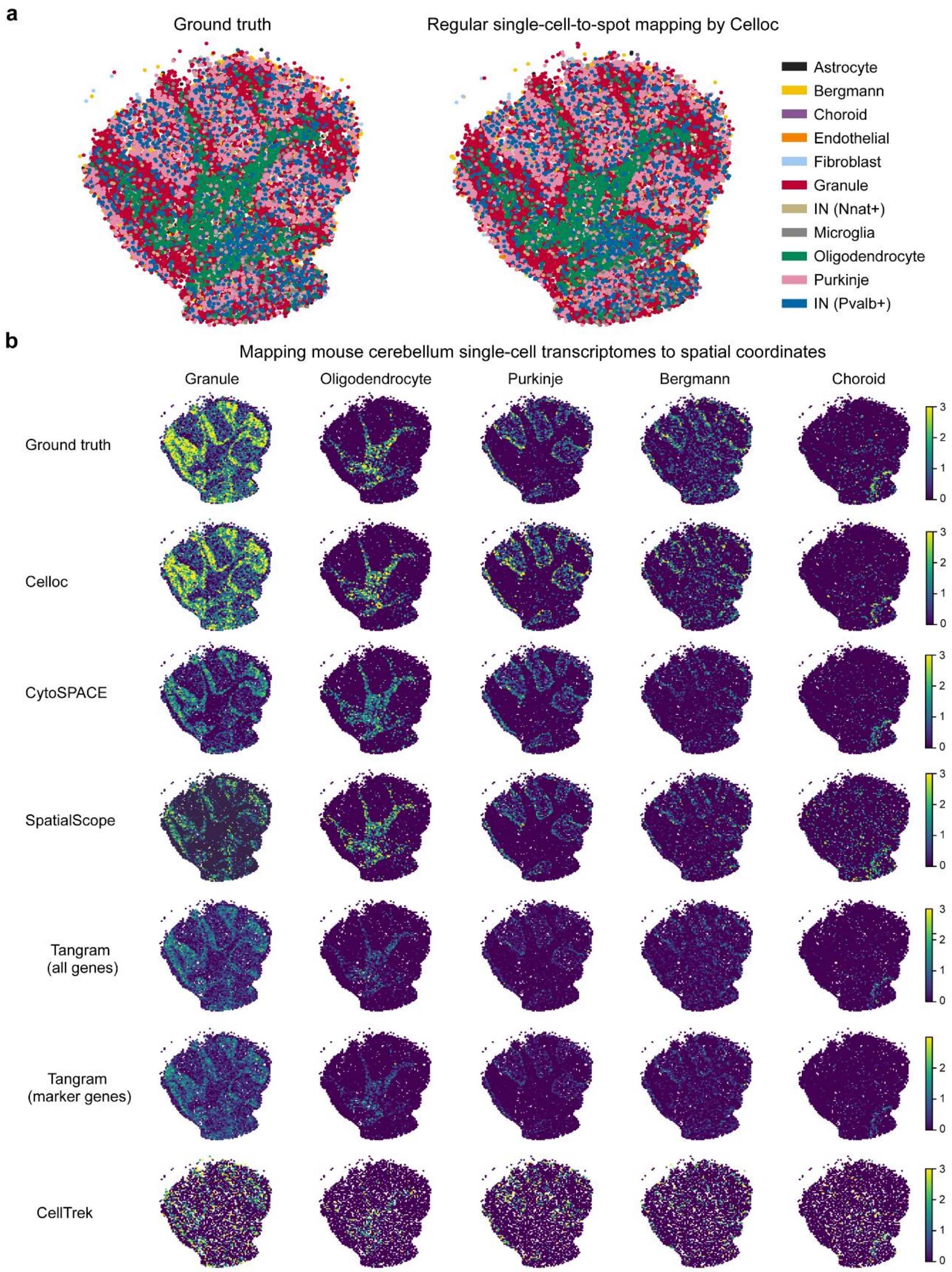

Supplementary Fig. 3. Visualization of regular mapping results on simulated cerebellum datasets. The simulated scRNA-seq data with 5% noise level and ST data with a mean of 5 cells per spot were selected as the visualization example. **(a)** The ground truth and regular mapping results by Celloc. **(b)** The ground truth and regular mapping results by distinct methods for granule cells, oligodendrocytes, Purkinje neurons, Bergmann glia, and Choroid cells, respectively.

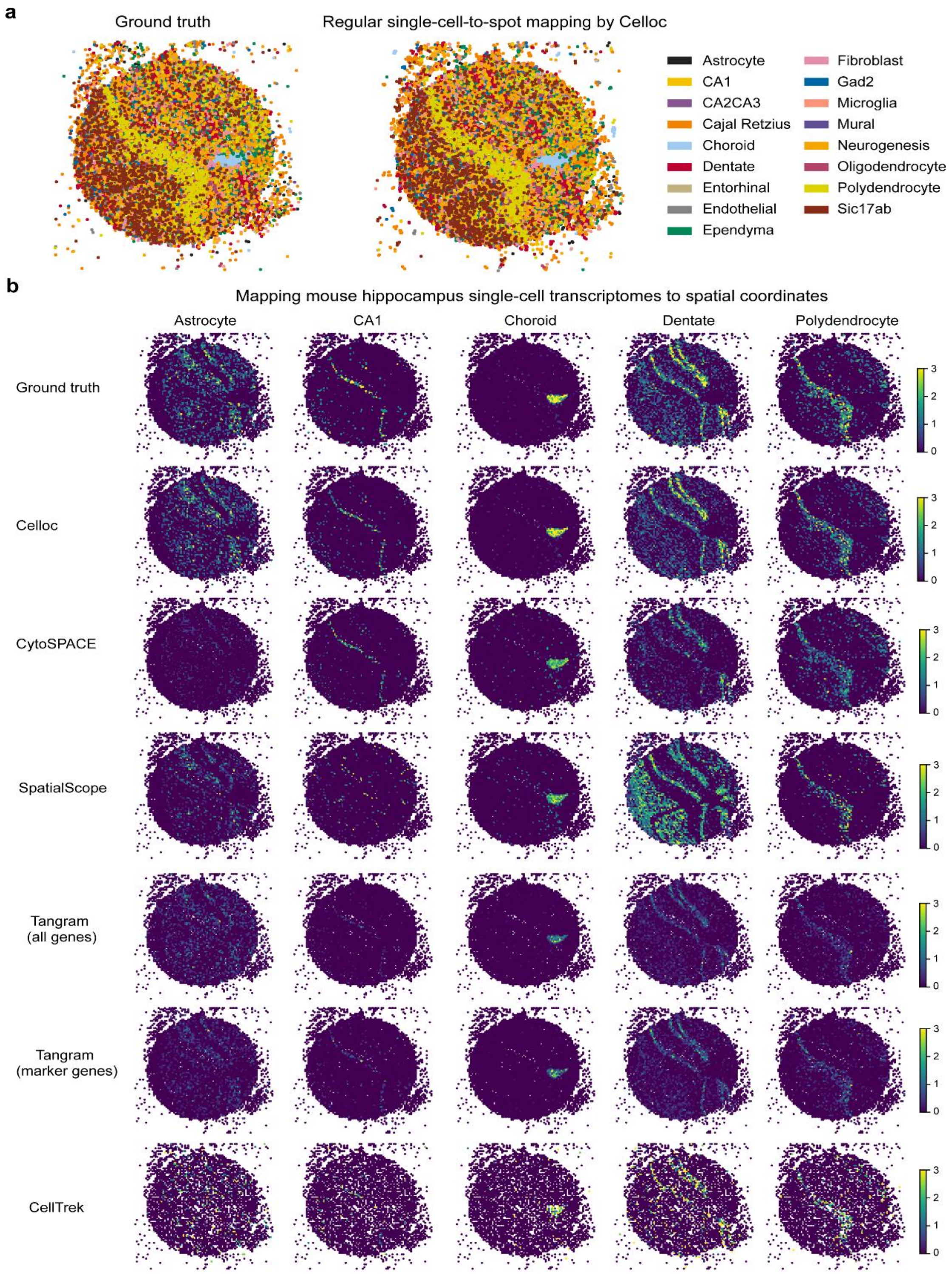

Supplementary Fig. 4. Visualization of regular mapping results on simulated hippocampus datasets. The simulated scRNA-seq data with 5% noise level and ST data with a mean of 5 cells per spot were selected as the visualization example. **(a)** The ground truth and regular mapping results by Celloc. **(b)** The ground truth and regular mapping results by distinct methods for astrocytes, CA1, Choroid cells, dentate gyrus, and polydendrocytes, respectively.

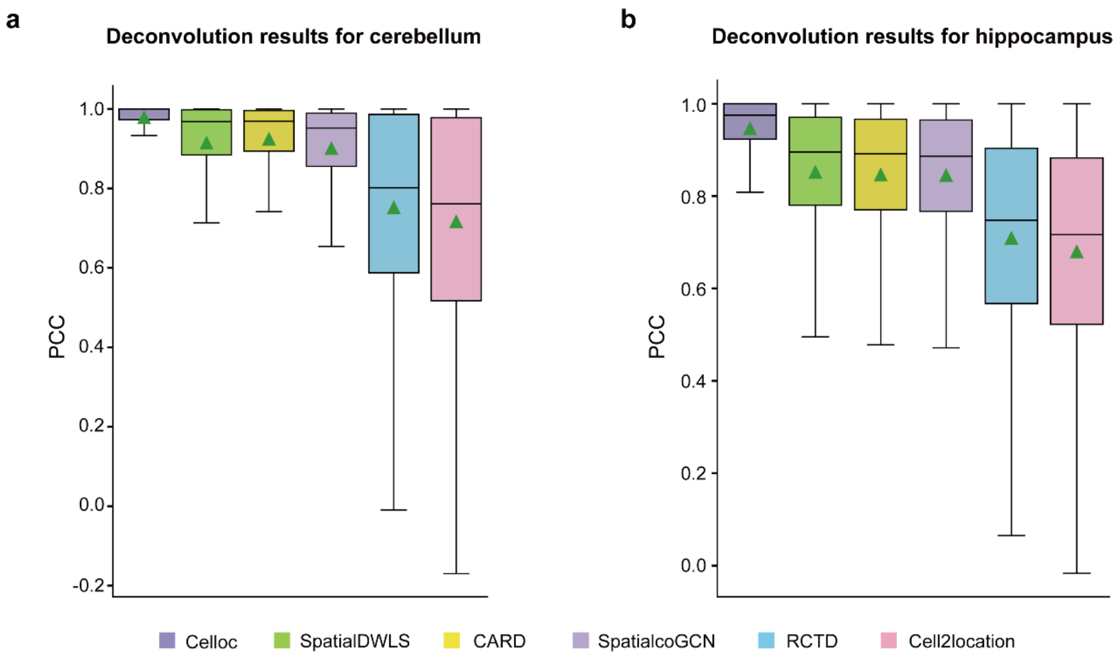

Supplementary Fig. 5. The performance of Celloc and traditional deconvolution methods in deconvolution. (a) The deconvolution results for the simulated cerebellum dataset. **(b)** The deconvolution results for the simulated hippocampus dataset.

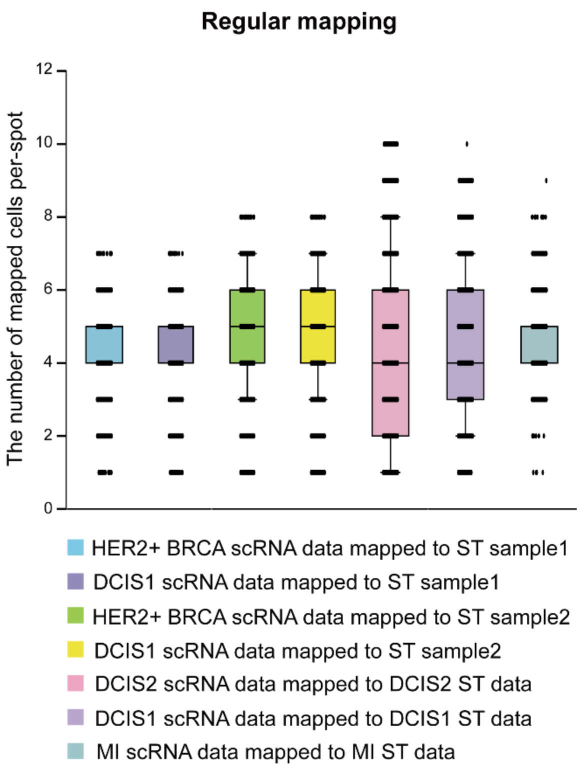

Supplementary Fig. 6. The actual number of cells mapped to each spot in each mapping case.

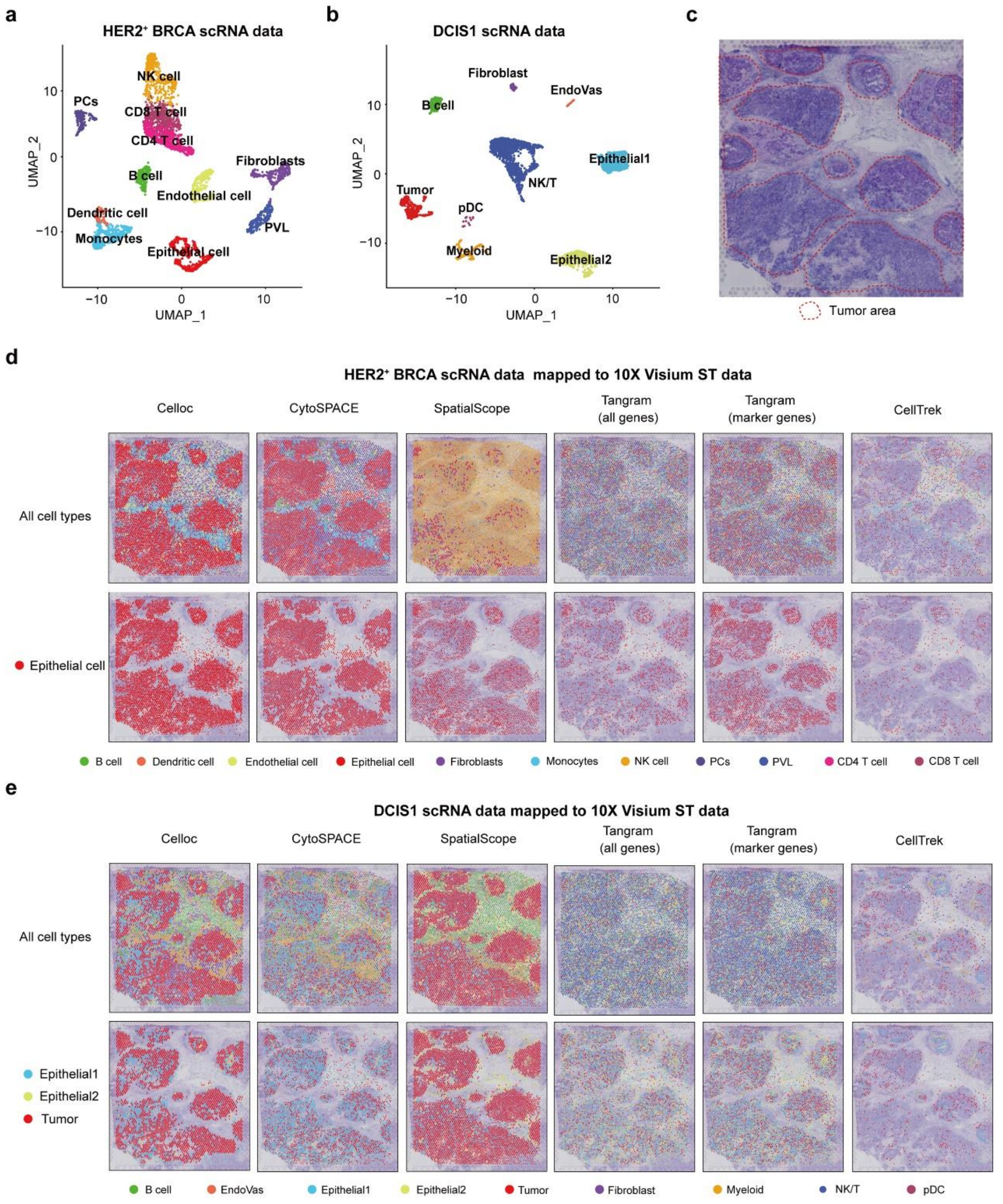

Supplementary Fig. 7. Visualization of (regular) mapping results of breast cancer ST dataset (sample2) with different scRNA-seq references. To assess the robustness of methods toward different scRNA-seq references, two alternative scRNA-seq datasets, i.e. HER2$^+$ BRCA and DCIS1 scRNA-seq datasets, were used as the reference mapped to the 10X Visium breast cancer ST sample2. **(a)** UMAP of HER2$^+$ BRCA scRNA-seq data, showing 11 cell types, including epithelial cells but not tumor cells. **(b)** UMAP of DCIS1 scRNA-seq data, showing 9 cell types including tumor cells and two types of epithelial cells. **(c)** H&E image of the 10X Visium ST sample1 with five different labeled regions. **(d)** Visualization of the mapping results by Celloc and the previous methods for all cell types and epithelial cells, using the HER2$^+$ BRCA scRNA-seq data and ST sample2 data. **(e)** Visualization of the mapping results by Celloc and the

previous methods for all cell types and epithelial 1/2 subgroups, using the DCIS1 scRNA-seq data and ST sample2 data.

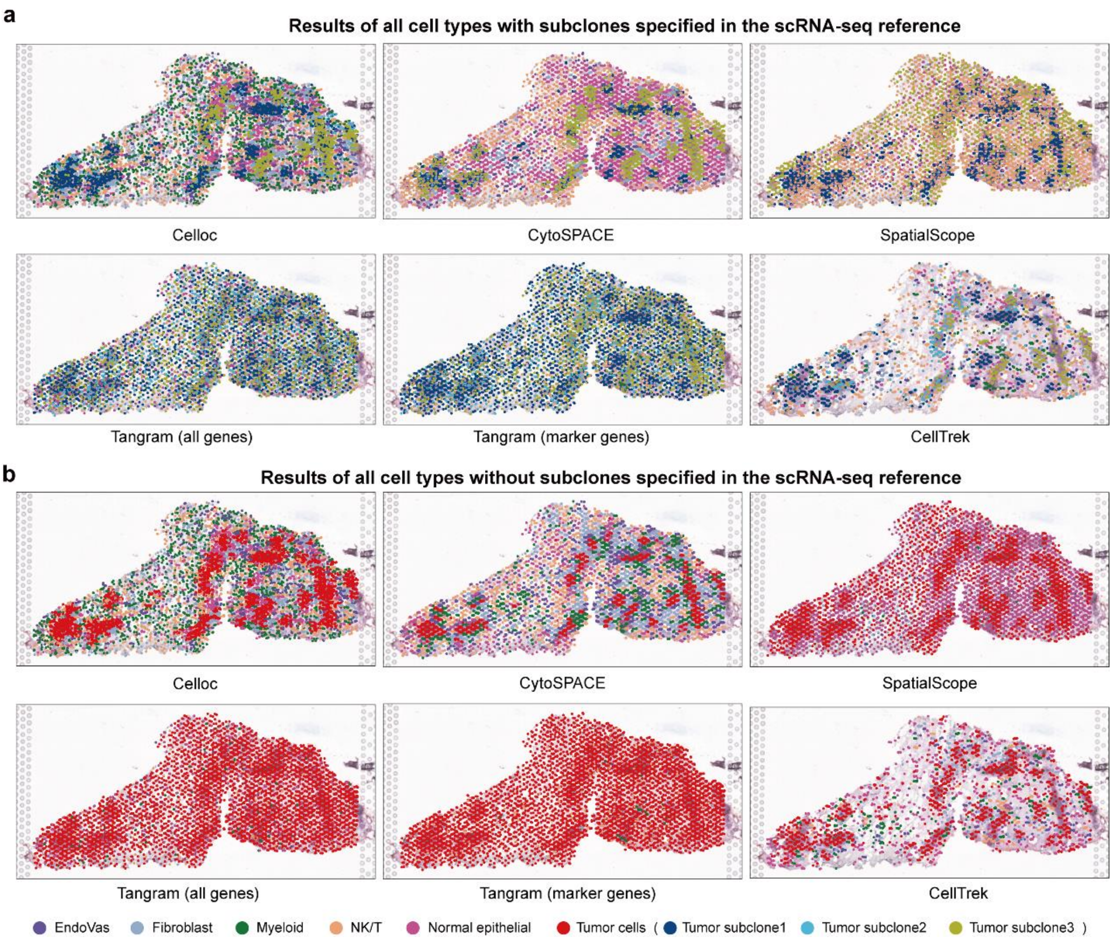

Supplementary Fig. 8. Visualization of (regular) mapping results of all cell types in DCIS dataset with and without subclone type specified in scRNA-seq reference. (a) Visualization of the mapping results of all cell types with subclones specified in the scRNA-seq reference. (**b**) Visualization of the mapping results of all cell types without subclones specified in the scRNA-seq reference.

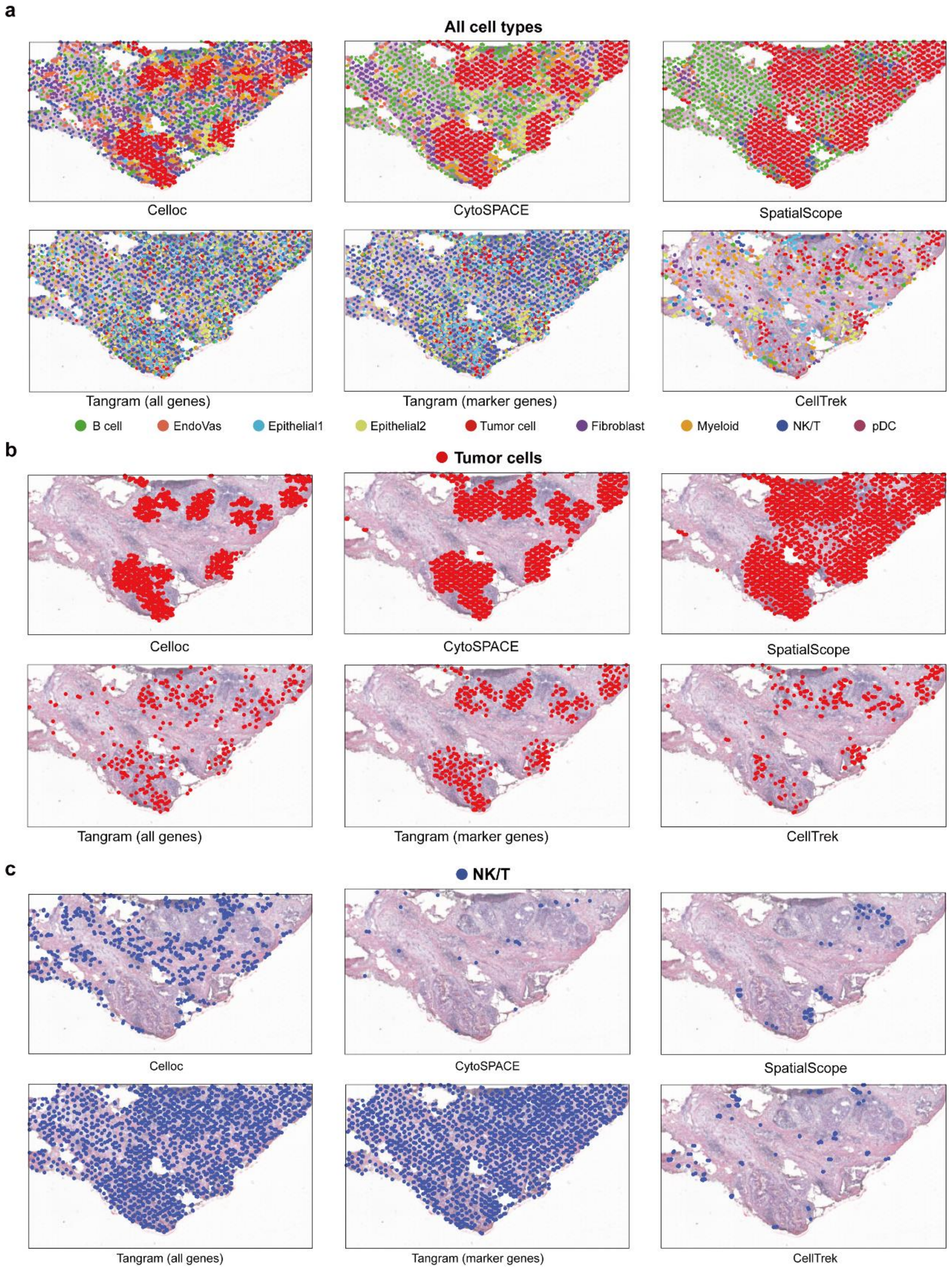

Supplementary Fig. 9. Visualization of (regular) mapping result by Celloc and the previous methods on the dataset for tumor-immune microenvironment analysis. (a) The mapping results of all cell types. **(b)** The mapping results of tumor cells. **(c)** The mapping results of NK/T cells.

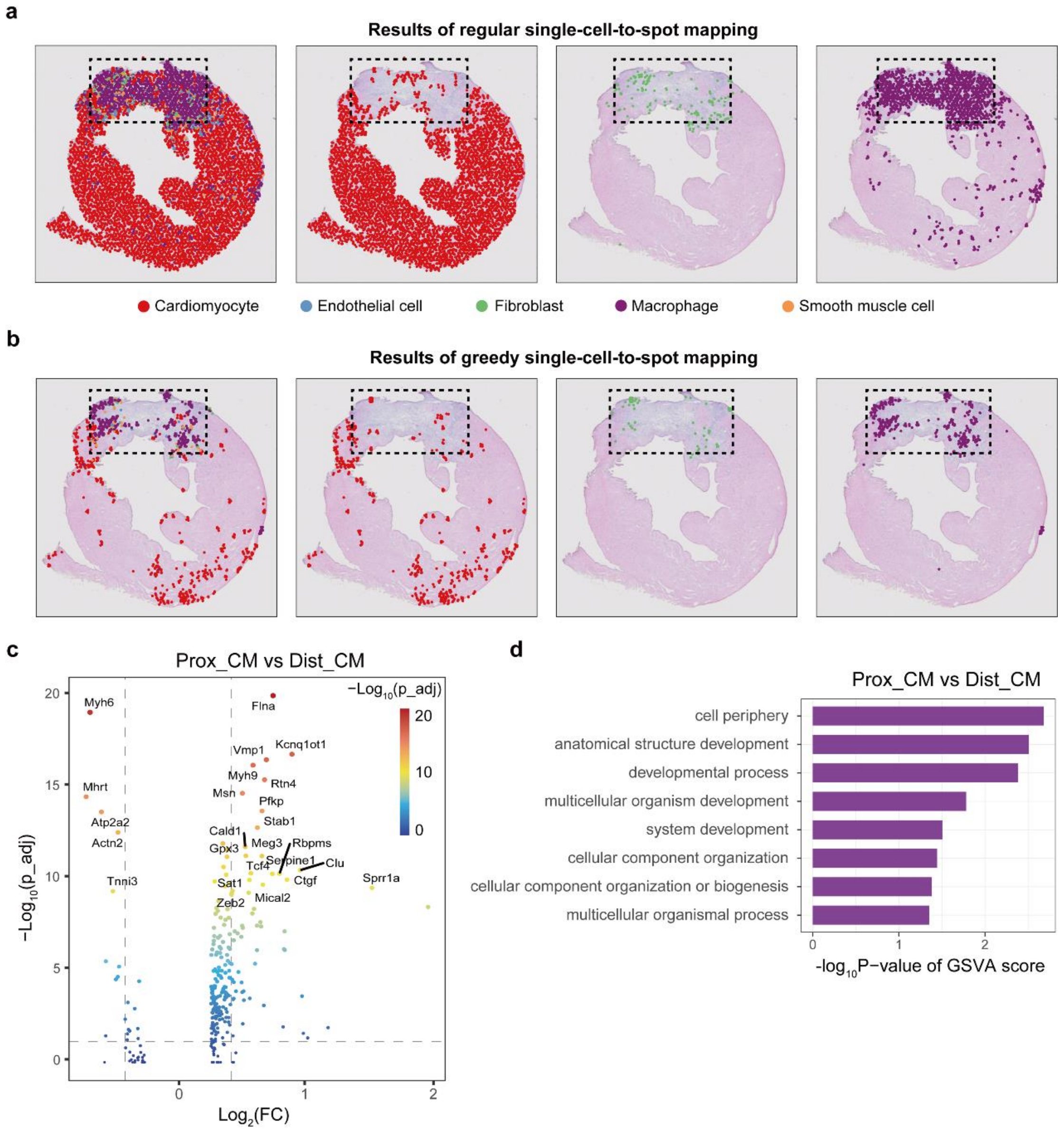

Supplementary Fig. 10. The spatial distribution and gene spatial expression patterns in myocardial infarction (MI) derived from Celloc mapping results. (a) Visualization of the spatial distribution of different cardiac cell types in the regular mapping result. The dotted box represents the infarcted area. **(b)** Visualization of the spatial distribution of different cardiac cell types in the greedy mapping result. **(c)** Differentially expressed genes between cardiomyocytes group proximal (Prox_CM) and distal (Dist_CM) to the fibrotic infarct area. More specifically, K-distance from cardiomyocytes to fibroblasts was calculated to group the cardiomyocytes based on the greedy mapping result. **(d)** Pathways showing high activities among the Prox_CM group in GSVA analysis.

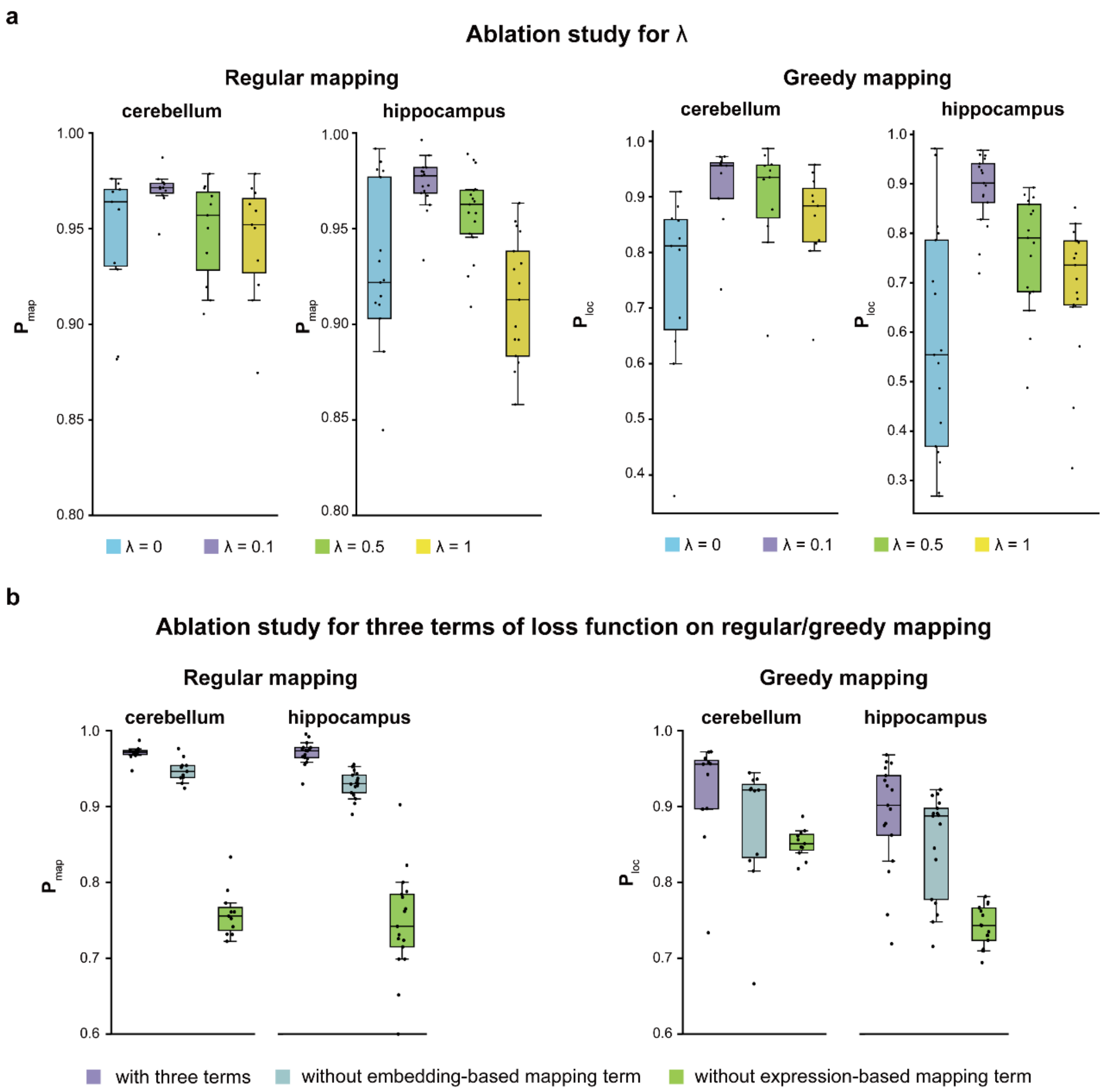

Supplementary Fig. 11. Performance of Celloc using different term weight parameter. To control overfitting, only the simulated scRNA-seq data with 5% noise level and ST data with a mean of 5 cells per spot were used for the selection of term weight parameter. **(a)** The parameters λ control the weight of cell quantity constraint term in the objective function. The boxplots of regular/greedy mapping results across different λ values on simulated cerebellum and hippocampus datasets. **(b)** The ablation study for three terms of loss function on regular/greedy mapping.
